# Silent controllers: non-coding RNAs drive autophagic response in myocardial ischemia reperfusion injury

**DOI:** 10.3389/fcvm.2026.1889759

**Published:** 2026-09-10

**Authors:** Afifa Azhar, Suhail Al-Salam

**Affiliations:** 1 Department of Pathology, College of Medicine & Health Sciences, United Arab Emirates University, AlAin, UAE; 2 Zayed Center for Health Sciences, United Arab Emirates University, AlAin, UAE

**Keywords:** autophagy, heart, ischemia-reperfusion injury, myocardial injury, non-coding RNAs

## Abstract

Acute myocardial infarction (AMI) continues to be a major contributor to global illness and death. Restoring coronary blood flow is a key strategy for limiting myocardial damage after infarction. Nevertheless, the re-establishment of circulation can paradoxically trigger ischemia–reperfusion (IR) injury through multiple intricate mechanisms. Consequently, IR injury remains an important obstacle in the effective management of myocardial infarction. Aberrant autophagy represents a major factor causing IR-related cardiomyocyte death. Autophagy, a catabolic process essential for cellular homeostasis, plays a bimodal role in myocardial IR —protective during ischemia yet potentially deleterious during reperfusion. Recent research highlights the pivotal regulatory function of non-coding RNAs (ncRNAs) which include microRNAs (miRNAs), long non-coding RNAs (lncRNAs), and circular RNAs (circRNAs) in modulating autophagy during myocardial IR injury, particularly in murine models. ncRNAs influence key autophagy regulators such as Beclin-1, ULK1, ATG5, ATG7, ATG12, LC3 and mTOR, forming complex regulatory networks that determine cardiomyocyte fate. This review synthesizes current evidence on how ncRNAs orchestrate autophagy in the setting of myocardial IR injury, elucidating underlying mechanisms and translational potentials. Understanding ncRNA-mediated modulation of autophagy opens avenues for targeted interventions to ameliorate IR injury related cardiac damage.

## 1 Introduction

In today's modern era, AMI remains a major global cause of both morbidity and mortality (1). The primary underlying mechanism of AMI is myocardial ischemia resulting from impaired coronary blood flow, commonly caused by coronary thrombosis, arterial stenosis, or vasospasm, which creates an imbalance between myocardial oxygen supply and demand (2, 3). Myocardial ischaemia can be managed with thrombolytic therapy or percutaneous coronary intervention to restore blood flow to the heart (2). Nevertheless, these therapeutic interventions may induce IR injury in the myocardium through several complex mechanisms, including intracellular ions overload, loss of mitochondrial membrane integrity, and excessive generation of reactive oxygen species (1). Myocardial IR injury is currently a key factor in the development of cardiovascular disease (4). It is involved in multiple pathological mechanisms, such as cardiomyocyte death via apoptosis, autophagy, and necrosis, as well as inflammation, oxidative stress, and mitochondrial dysfunction, all of which contribute to the onset and progression of IR injury (3, 4). One of the processes that gets activated in response to IR injury is autophagy (1). The large amount of generated reactive oxygen species (ROS) significantly increases autophagy (1). An excessive activation of this process is commonly associated with enhanced autophagic and apoptotic loss of cardiomyocytes (2–5). Growing evidence has shown that autophagy plays an important role in myocardial IR injury (2, 3, 6). Autophagy is a highly conserved cellular process throughout evolution that plays a vital role in maintaining cellular homeostasis. It has dual role in IR injury: protective during ischemia by preserving energy and removing damaged components, yet detrimental during reperfusion if excessively activated (2, 3, 6). Although modulation of autophagy is considered important in cardiovascular disorders, effective therapies targeting autophagy-related cardiac diseases and heart failure remain unavailable. Increasing evidence indicates that ncRNAs have significant regulatory roles in multiple biological pathways involved in myocardial IR injury (2, 3, 7). Therefore, investigating the molecular pathways through which non-coding RNAs regulate autophagy may offer promising therapeutic targets and novel intervention strategies for cardiovascular diseases (7, 8).

## 2 Methodology

This systematic review was conducted to evaluate the role of ncRNAs in autophagy during myocardial IR injury. To ensure methodological rigor, transparency, and reproducibility, a comprehensive literature search was independently conducted by two reviewers using the electronic databases PubMed/MEDLINE and Scopus from database inception to June 2026. To minimize the possibility of missing relevant publications, the reference lists of eligible original articles and relevant review papers were manually screened to identify additional studies.

The search strategy combined Medical Subject Headings (MeSH) and free-text keywords related to the three principal concepts of this review: ncRNAs, autophagy, and myocardial IR injury. The PubMed search strategy was constructed as follows:

(“non-coding RNA” OR “noncoding RNA” OR ncRNA OR microRNA OR miRNA OR lncRNA OR “long non-coding RNA” OR circRNA OR “circular RNA”) AND (autophagy OR mitophagy) AND (“myocardial ischemia reperfusion injury” OR “myocardial ischemia-reperfusion injury” OR “myocardial IR injury” OR cardiac ischemia reperfusion”).

An equivalent search strategy was adapted for the Scopus database using Boolean operators (AND/OR), phrase searching, and database-specific indexing terms to maximize both sensitivity and specificity.

**Figure 1:**
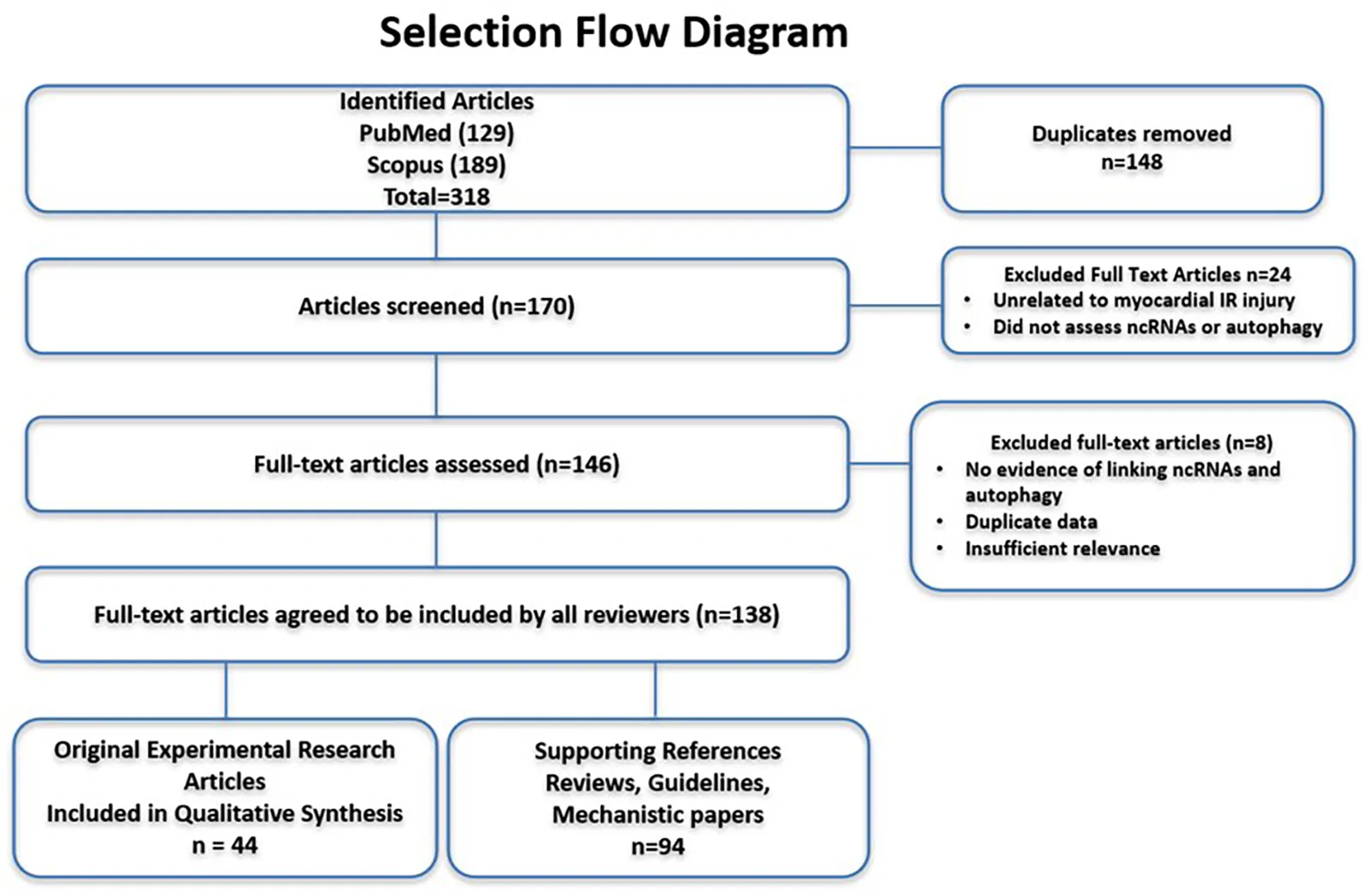
Selection flow diagram illustrating the identification, screening eligibility and final includes articles that studied myocardial IR injury, autophagy and ncRNAs.

All retrieved citations were exported into EndNote X20 (Clarivate Analytics) for reference management, and duplicate records were removed through both automated and manual screening. The initial electronic search yielded 318 records (PubMed = 129; Scopus = 189). After duplicate removal, 170 unique publications remained for title and abstract screening. During this stage, 24 articles were excluded because they were unrelated to myocardial IR injury, did not evaluate non-coding RNAs or autophagy. Consequently, 146 full-text articles were retrieved and assessed for eligibility. Following full-text evaluation, 8 articles were excluded because they lacked sufficient mechanistic evidence linking non-coding RNAs with autophagy, contained duplicate experimental data, or failed to show sufficient relevance. Only 138 articles were selected, of those 44 articles were Original experimental studies meeting the predefined eligibility criteria while the other 94 articles were considered as supporting references. The study selection process is summarized in Figure 1.

The predefined inclusion criteria comprised: (i) original peer-reviewed research articles; (ii) studies investigating myocardial ischemia-reperfusion injury using *in vivo* animal models, *in vitro* hypoxia/reoxygenation models, or *ex vivo* cardiac preparations alone or in relation to ncRNA, (iii) studies evaluating the role of miRNAs, ln RNAs, circular RNAs, circRNAs, or other regulatory ncRNAs in autophagy during myocardial IR injury; and (iv) studies investigating autophagy, mitophagy, autophagic flux, or autophagy-related signalling pathways in the context of myocardial ischemia-reperfusion injury. Only articles published in English with full-text availability were considered eligible.

The exclusion criteria included studies unrelated to myocardial ischemia-reperfusion injury or autophagy or ncRNAs, publications lacking experimental evidence regarding the interaction between non-coding RNAs and autophagy, conference abstracts, editorials, letters, case reports, book chapters, duplicate publications, and studies with insufficient methodological or experimental information.

Data extraction was independently performed by two reviewers using a standardized data extraction form. Extracted information from original experimental research articles included the first author, publication year, country of origin, experimental model, sample size, investigated non-coding RNA, autophagy-associated molecular targets, signalling pathways, experimental techniques, and principal findings. While Extracted information from supporting articles included up-to-date background data on autophagy, myocardial IR injury, miRNAs, lncRNAs, circRNAs, guidelines, mechanisms of interaction, development of biomarkers, diagnostic and therapeutic prospectives. Any disagreements between reviewers were resolved through discussion until consensus was achieved.

The findings were synthesized using a qualitative narrative approach, emphasizing the molecular mechanisms through which non-coding RNAs regulate autophagy and influence myocardial ischemia-reperfusion injury.

## 3 Autophagy

In 1963, Christian de Duve was first to introduce autophagy (self-eating) as a lysosome-mediated disposal process (9). Autophagy is a degradative cellular mechanism responsible for eliminating damaged organelles and accumulated protein aggregates through lysosomal breakdown, thereby contributing significantly to the preservation of cellular homeostasis (9–11). Primarily, it maintains cellular equilibrium by coordinating organelle turnover, protein production, and the removal of damaged cellular components. Autophagy is commonly activated in response to conditions such as nutrient deficiency, oxidative stress, hypoxia, and exposure to toxic foreign substances (9–11).

It can be classified as macroautophagy, microautophagy and chaperone-mediated autophagy (CMA) reliant on their physiological roles and delivery routes (9, 10).

The process of macroautophagy unfolds in a series of distinct phases: initiation, nucleation, elongation/expansion, closure, and the ultimate fusion of phagosomes with lysosomes and degradation of autophagosomes (12–14). This intricate cellular mechanism begins with the genesis of a pre-autophagosomal structure, termed a phagophore, which can originate from various intracellular membranes such as the endoplasmic reticulum or mitochondria (13). These nascent phagophores migrate to specialized assembly sites, where they recruit essential components of the autophagy machinery (12–14). Subsequently, they undergo significant expansion, enveloping cytoplasmic material designated for degradation, thereby maturing into double-membraned autophagosomes (12–14). These fully formed autophagosomes are then actively transported along cytoskeletal networks, particularly microtubules, culminating in their delivery to lysosomes for proteolytic breakdown (12).

The intricate orchestration of autophagosome biogenesis is meticulously governed by a suite of autophagy-related genes (ATGs) (15–17). Genetic screens conducted in yeast have unveiled over thirty such genes, many of which possess conserved mammalian homologs. A pivotal regulatory step in phagophore induction and expansion involves the class-III phosphoinositide 3-kinase, Vps34. This enzyme forms a critical multiprotein complex with Beclin1, ATG14, and Vps150, leading to the localized production of phosphatidylinositol 3-phosphate (PtdIns3P) (16, 17). This lipid signal is indispensable for recruiting additional regulatory proteins to the phagophore assembly site, thereby facilitating phagophore elongation. Furthermore, phagophore initiation is also triggered by a macromolecular complex comprising ATG13, Unc-51-like kinase 1 (Ulk1, a mammalian ortholog of yeast ATG1), and Ulk2 (15–17). This complex phosphorylates the focal adhesion kinase-family interacting protein 200 (FIP200), a crucial event that promotes the onset of autophagy (15–17). The subsequent elongation of the phagophore is mediated by two ubiquitin-like conjugation systems. Initially, ATG12 is covalently linked to ATG5 through the enzymatic actions of ATG7 and ATG10. The resulting ATG12-ATG5 conjugate then associates with the phagophore membrane, driving its expansion (17). Following this, ATG7, ATG3, and the ATG12–ATG5 complex cooperate to mediate the conjugation of the cytosolic form of LC3 (ATG8), termed LC3-I, with phosphatidylethanolamine, resulting in the formation of the membrane-associated LC3-II. LC3-II is essential for autophagosome maturation and elongation, facilitating membrane tethering and hemifusion while also controlling autophagosome dimensions through regulation of membrane curvature (16, 17). Upon completion of autophagosome formation, LC3-II is liberated back into the cytosol following its Atg4-mediated cleavage from phosphatidylethanolamine. While the precise molecular mechanisms and genes governing autophagosome-lysosome fusion in mammals remain an active area of research, it is established that this event necessitates the lysosomal membrane protein LAMP-2 and the small GTPase Rab7 (15–17). Following autophagosome-lysosome fusion, the carried cargo by autophagosomes will be degraded by lysosomal enzymes (12, 13).

During microautophagy, cellular components are directly engulfed by the lysosomal membrane and subsequently degraded within the lysosome (18, 19). In CMA, specific chaperone proteins recognize and transport target proteins to lysosomes, where they undergo enzymatic degradation (18, 20). Among the different forms of autophagy, macroautophagy is the most extensively investigated and commonly occurring pathway; therefore, the term “autophagy” in this review refers specifically to macroautophagy. In addition to these classical forms, two novel pathways known as RNautophagy and DNautophagy have been described by Yuuki Fujiwara and colleagues (21–23). They described two unconventional lysosome-dependent degradation pathways, in which nucleic acids are transported directly into lysosomes rather than being delivered through conventional autophagosomes. RNautophagy mediates the direct lysosomal uptake and degradation of RNA, whereas DNautophagy performs an analogous function for DNA. Both pathways involve the lysosomal membrane protein LAMP2C, whose cytosolic tail can bind nucleic acids and facilitate their lysosomal uptake; the process is ATP dependent (22, 23). Nevertheless, the precise physiological significance of RNautophagy and DNautophagy in myocardial IR injury has not yet been fully elucidated (21).

### 3.1 Hypoxia and autophagy

Under hypoxic conditions, autophagy is a survival mechanism. hypoxia does not trigger cell death, but it triggers a survival process i.e., autophagy. In the context of hypoxia, autophagy plays a vital role in sustaining cellular ATP generation and macromolecule synthesis, thereby serving as an important cellular survival mechanism (24).

Hypoxia induces autophagy through an integrated network that couples oxygen sensing, energy stress, and growth signalling into a unified adaptive response. Central to this process is the stabilization of hypoxia-inducible factor-1*α* (HIF-1*α*) under low oxygen conditions, a master transcriptional regulator of cellular adaptation that promotes autophagy-related gene expression (25–27). Notable downstream targets include BNIP3 and NIX, both of which are induced under hypoxic conditions and stimulate autophagy by interfering with the suppressive interaction between Bcl-2 and Beclin-1. This disruption releases Beclin-1, enabling the initiation of autophagosome development (26, 27).

In parallel, reduced oxidative phosphorylation under hypoxia lowers ATP levels and elevates the AMP/ATP ratio, leading to activation of AMP-activated protein kinase (AMPK), a central energy sensor that promotes autophagy both by directly activating the ULK1 complex and by inhibiting mTORC1 signalling (26–28). This suppression of mTOR, a key negative regulator of autophagy, represents a critical convergence point through which hypoxic and metabolic signals induce autophagic flux (25–28).

Collectively, these pathways not only stimulate bulk autophagy but also drive selective mitophagy via BNIP3/NIX-mediated mitochondrial targeting, thereby reducing oxygen consumption and limiting reactive oxygen species production as part of an adaptive survival strategy under hypoxic stress (24–29).

During the reperfusion stage, AMPK activity becomes suppressed, while autophagy is triggered through increased expression of Beclin-1. The excessive production of reactive oxygen species during ischemia–reperfusion injury is considered a major contributor to the upregulation of Beclin-1 (25–30).

### 3.2 Oxidative stress and autophagy

Mitochondria represent the primary intracellular source of ROS, which are mainly generated as by-products of oxidative phosphorylation (OXPHOS) (9, 18, 24, 29, 30). At physiological levels, ROS function as important signalling molecules involved in numerous cellular activities, including cell proliferation, differentiation, programmed cell death, innate immune responses, autophagy, redox regulation, calcium balance, adaptation to hypoxia, and stem cell reprogramming (9, 18, 24, 29, 30). However, excessive oxidative stress can damage proteins and other cellular structures, contributing to the development of multiple pathological conditions (9). Under normal conditions, ROS-mediated signalling can stimulate autophagy to preserve cellular homeostasis, whereas disruption of redox balance may impair autophagic function and promote disease progression (9, 18, 24). Mitochondria act both as producers and targets of ROS, making them central mediators of oxidative stress (9, 29). Persistent oxidative stress leads to oxidation and injury of essential cellular components, including proteins, lipids, and DNA, which subsequently activate autophagic pathways (9, 18, 29). Cardiomyocytes are particularly rich in mitochondria, and damaged or dysfunctional mitochondria are selectively eliminated through mitophagy (9, 29). Defects in this degradative process result in excessive accumulation of mitochondrial ROS, aggregation of damaged proteins, mitochondrial dysfunction, and adverse cardiac remodelling. Autophagy has therefore been strongly linked to the initiation and progression of various cardiovascular disorders, including IR injury (9, 18, 30). By eliminating damaged mitochondria and other cellular components that produce ROS, autophagy has been demonstrated to contribute to the reduction of oxidative stress (9, 24). Enhancing autophagy may be a therapeutic approach for oxidative stress-related diseases like neurodegenerative disorders, cardiovascular diseases, and some types of cancer since it can help restore cellular homeostasis and lessen oxidative damage (9, 29). Nevertheless, depending on the cellular and tissue context, autophagy may also aggravate disease progression in response to mitochondrial ROS generation (9, 18, 24, 29). Therefore, mitochondrial ROS-induced autophagy can function either as a protective adaptive mechanism that reduces oxidative stress or as a harmful process that contributes to cellular injury (9).

### 3.3 Autophagy in the heart during ischemia and IR injury

Autophagy is an evolutionarily conserved intracellular degradation pathway that plays a fundamental role in maintaining cardiac homeostasis by removing damaged proteins, dysfunctional mitochondria, and other injured cellular components through lysosome-dependent degradation (10, 30–33). Under physiological conditions, basal autophagy preserves cardiomyocyte integrity and energy metabolism, whereas dysregulation of this process contributes to the development and progression of cardiovascular diseases (10, 30–33). Moderate activation of autophagy generally promotes cell survival by maintaining protein quality control and metabolic adaptation, while either insufficient or excessive autophagy may result in cellular dysfunction and death (10, 36).

**Figure 2:**
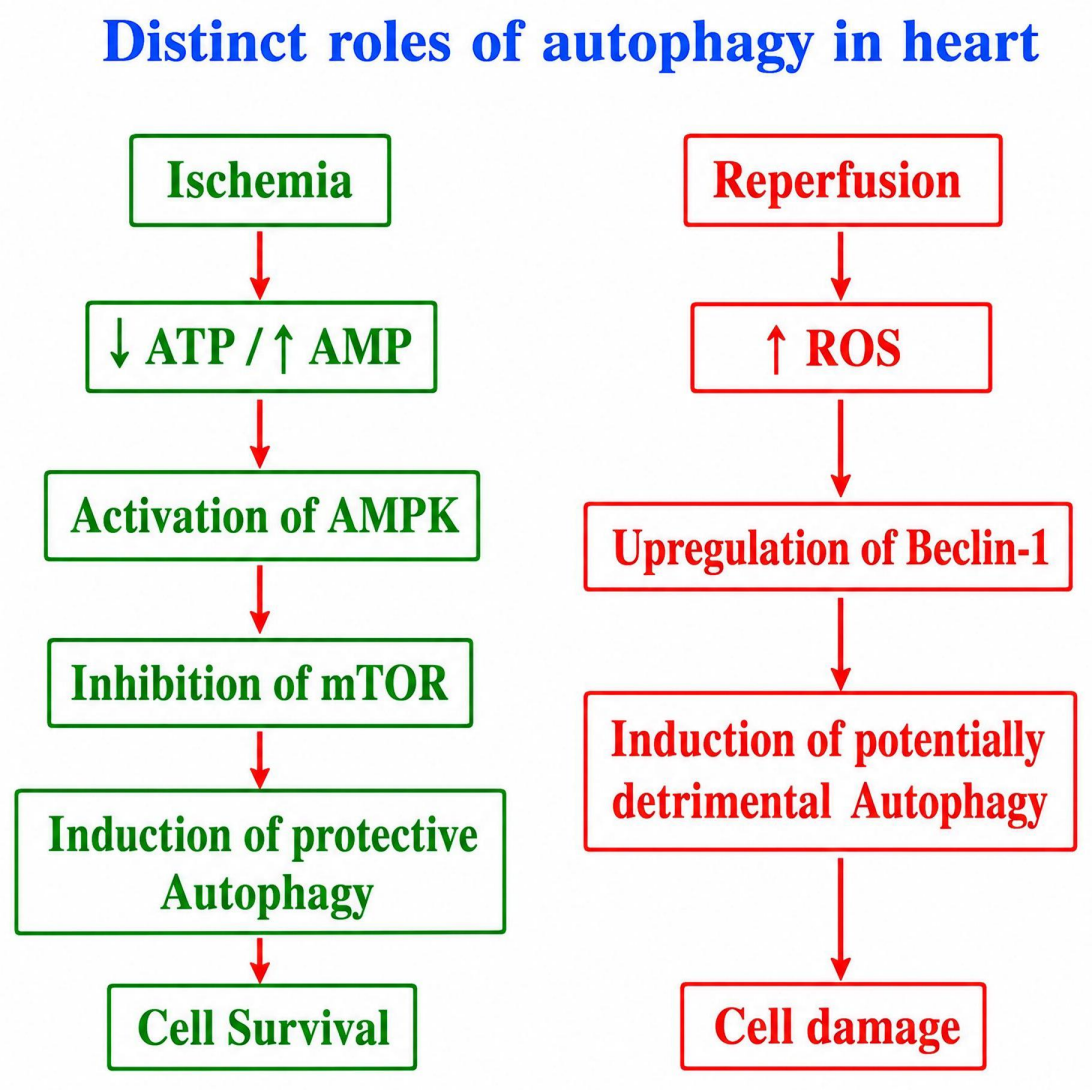
Illustrating the distinct roles of autophagy in the heart during ischemia and reperfusion injury.

The role of autophagy during myocardial IR injury is highly dynamic and depends on the timing, magnitude, and duration of activation (6, 32, 35). During ischemia (Figure 2), nutrient deprivation and ATP depletion activate AMPK, which suppresses mTOR and induces a moderate level of autophagy (6). This adaptive response provides metabolic substrates through intracellular recycling, removes damaged mitochondria before they generate excessive ROS, and delays apoptotic and necrotic cell death (32–34). Consequently, ischemia-induced autophagy is generally regarded as cardioprotective (6, 32–34).

In contrast, reperfusion induces oxidative stress (Figure 2), calcium overload, mitochondrial dysfunction, inflammation, and endoplasmic reticulum stress, activating autophagy predominantly through Beclin-1-dependent but AMPK-independent mechanisms (6, 30, 35). Unlike ischemic autophagy, excessive or sustained activation during reperfusion may promote degradation of essential cellular constituents, aggravate mitochondrial injury, and contribute to cardiomyocyte death (6, 30, 35). Therefore, autophagy has been widely described as a “double-edged sword” during myocardial IR injury (32, 35).

However, the relationship between autophagy and myocardial injury is more complex than simply classifying ischemic autophagy as beneficial and reperfusion autophagy as harmful. Increasing evidence indicates that the degree of autophagy activation is critical (30, 35, 37). Moderate activation maintains mitochondrial quality control, limits oxidative stress, and preserves cellular bioenergetics (34, 36, 37), whereas excessive or prolonged autophagy may induce autophagy-associated cell death or potentiate apoptosis (30, 35). Conversely, complete inhibition of autophagy is also detrimental because damaged mitochondria and misfolded proteins accumulate, resulting in increased ROS generation, inflammasome activation, and impaired myocardial recovery (35–37). Therefore, maintenance of autophagic homeostasis, rather than indiscriminate activation or inhibition, appears to be essential for optimal cardioprotection (35, 36).

Another important consideration is autophagic flux, which represents the entire autophagic process from autophagosome formation to lysosomal degradation and recycling (3, 38). Many experimental studies assess autophagy by measuring LC3-II, Beclin-1, ATG proteins, or p62/SQSTM1 (3, 38). Although these markers are useful indicators, changes in their abundance alone cannot distinguish between increased autophagosome formation and impaired degradation (38). For example, elevated LC3-II levels may indicate either enhanced autophagy initiation or accumulation of undegraded autophagosomes resulting from defective autophagosome-lysosome fusion (3, 12, 38). Likewise, increased Beclin-1 expression reflects activation of the autophagy initiation machinery but does not necessarily indicate successful completion of the autophagic process (38). Current guidelines therefore recommend assessment of autophagic flux, including LC3 turnover in the presence of lysosomal inhibitors, tandem fluorescent LC3 reporter assays, and p62 degradation, to accurately determine whether autophagy is truly activated or impaired (3, 12, 38).

Lysosomal function is another essential determinant of effective autophagy (3, 4, 38). During reperfusion, oxidative stress and calcium overload may impair lysosomal acidification, decrease lysosomal enzyme activity, and inhibit autophagosome-lysosome fusion (8, 10, 12). Under these conditions, autophagosomes accumulate despite reduced degradative capacity, leading to defective autophagic flux and worsening myocardial injury (Figure 2) (8, 10). Thus, preservation of lysosomal integrity may be as important therapeutically as modulation of autophagy initiation itself (4, 30, 38).

Moreover, the consequences of autophagy differ among cardiac cell types (4, 36). Most mechanistic studies have focused on cardiomyocytes; however, endothelial cells, fibroblasts, macrophages, vascular smooth muscle cells, and infiltrating immune cells exhibit distinct autophagic responses during myocardial IR injury (4, 36). In cardiomyocytes, autophagy primarily regulates mitochondrial quality control and ATP production, whereas in endothelial cells it maintains endothelial integrity, nitric oxide bioavailability, and microvascular perfusion (4, 36). In cardiac fibroblasts, autophagy influences extracellular matrix turnover and post-infarction fibrosis, while macrophage autophagy regulates inflammatory responses and resolution of tissue injury (4, 36). These cell-specific differences help explain why modulation of a single autophagy-related pathway can produce divergent outcomes depending on the experimental model and timing of intervention (4, 36).

Collectively, current evidence indicates that the cardioprotective or detrimental effects of autophagy during myocardial ischemia/reperfusion injury depend on a complex interplay among the timing of activation, the magnitude of the autophagic response, preservation of autophagic flux, lysosomal competence, and cell type-specific functions (4, 6, 35, 36, 38). Therefore, therapeutic strategies should aim to restore balanced autophagic homeostasis rather than indiscriminately stimulating or suppressing autophagy (32, 35, 36). This concept is particularly important when interpreting studies investigating autophagy-regulating non-coding RNAs, as many reports rely primarily on static markers such as LC3-II or Beclin-1 without directly assessing autophagic flux, potentially leading to overinterpretation of autophagy activation or inhibition (3, 38).

## 4 Non-Coding RNAs

**Figure 3:**
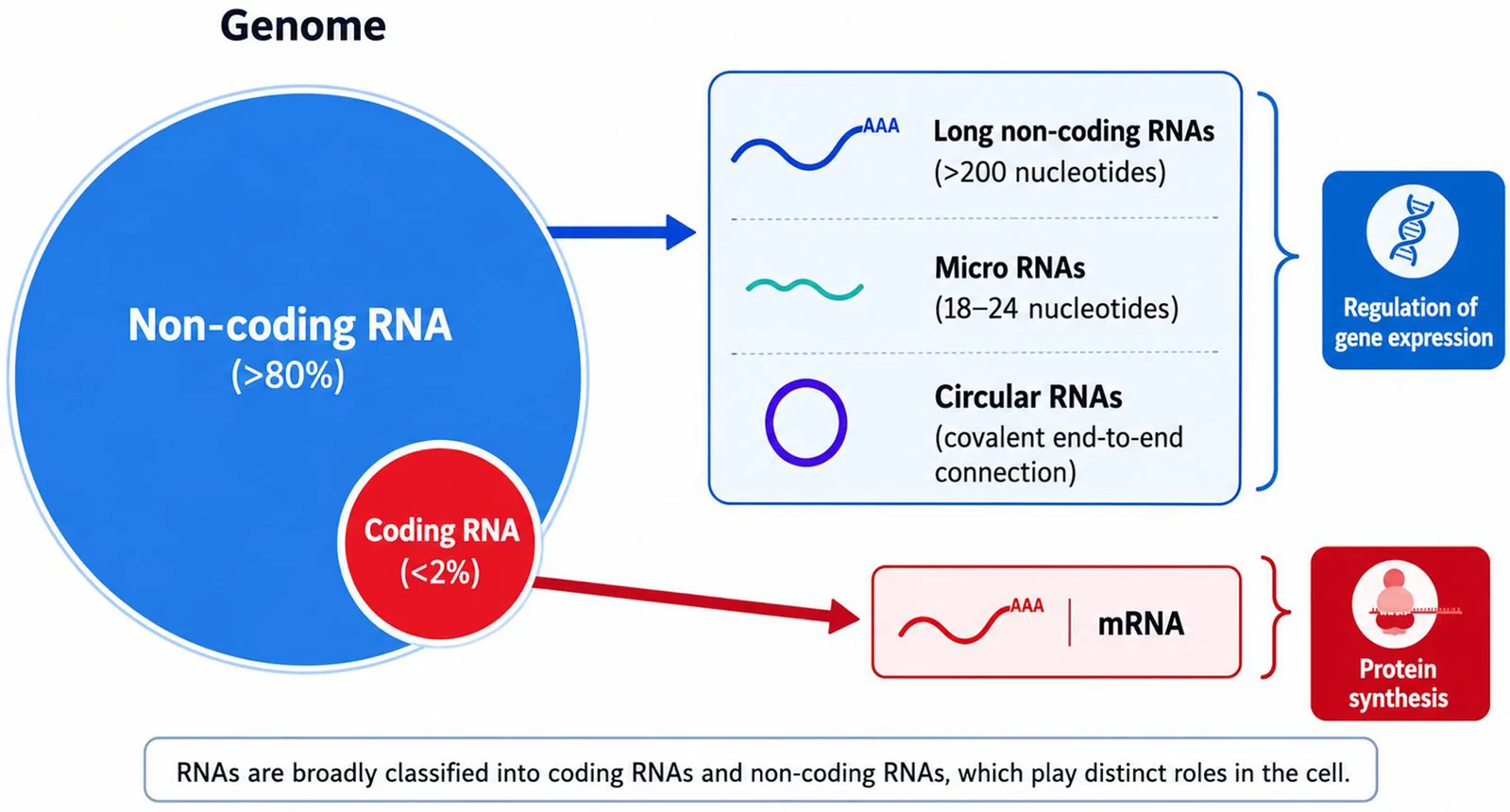
Illustrating the coding and non-coding RNAs in human genome.

The fundamental mechanism governing the transmission of genetic data, known as the central dogma of molecular biology, outlines the progression from deoxyribonucleic acid (DNA) to ribonucleic acid (RNA) and subsequently to protein, thereby establishing the core principles of gene expression (39, 40). While a substantial two-thirds of the human genome undergoes transcription, less than two percent of this transcribed material codes for proteins (Figure 3) (4, 39, 40). The untranslated portions are consequently processed into non-coding RNAs (ncRNAs). RNAs are broadly classified based on their capacity to encode proteins (coding or non-coding) and their specific biological roles, such as protein synthesis or the modulation of gene expression (Figure 3) (40, 41). Messenger RNA (mRNA) typically represents coding RNA, responsible for protein production, whereas ncRNAs function as cellular orchestrators without directly translating into proteins (39, 40). Historically, a significant fraction of the genome was dismissed as “junk” due to an incomplete understanding of its purpose (39, 40). However, the successful completion of the Human Genome Project (HGP) illuminated the critical importance of these previously enigmatic genomic segments, revealing their indispensable roles in vital biological processes (39–42). These non-coding regions within the human genome have since been found to exert profound regulatory influences across a diverse array of cell types, including neural, immune, and cardiovascular cells (42, 43). Advanced next-generation sequencing (NGS) technologies have facilitated the identification of thousands of these ncRNAs (42–44). These pervasive RNA molecules are instrumental in fine-tuning molecular pathways that profoundly impact numerous cellular functions and disease pathologies (43, 44). Present in virtually every tissue and organ, many ncRNAs are pivotal in the initiation and advancement of various diseases (42). Furthermore, an accumulating body of research indicates that ncRNAs play essential roles in various biological events occurring during myocardial IR injury, including processes like autophagy (7, 10, 45).

The ncRNAs were first described by Lee et al. in 1993 (45). Their expression is significantly changed in various cardiac diseases as an adaptive regulator of the body (45, 46). They are present in various cell types within the heart, including cardiomyocytes, cardiac fibroblasts, endothelial cells, immune cells and smooth muscle cells (45–47). Consequently, ncRNAs are defined as RNA species that maintain functional conservation despite lacking the capacity for protein translation; rather, they serve to regulate genetic expression through both transcriptional and post-transcriptional mechanisms (48).

ncRNAs are involved in regulating gene transcription and translation, RNA processing and modification, and chromosomal replication (39, 45). ncRNAs can also affect chromosome structure and dynamics, though they do not directly cause chromosome translocations like a protein might. ncRNAs can work at different stages of gene expression, including controlling transcription initiation, mRNA processing, and the efficiency of translation. ncRNAs can help maintain chromatin structure and keep the genome stable. They can also be influenced by changes in chromosomes (39, 40). Some fusion proteins that come from translocations can change ncRNA expression (39, 45). Many studies have indicated that ncRNAs, play important roles in the progress of myocardial IR injury through the modulation of autophagy in cardiomyocytes (39, 42). In myocardial IR injury, several factors can stimulate the production of ncRNAs including hypoxia, ROS, inflammatory signals, cellular stress pathways, and mechanical stretch (35, 45, 49). These ncRNAs play important regulatory roles in autophagy-related mTOR signalling pathways and the process of autophagosome formation (10, 35). It is established that majority of ncRNAs are prominently dysregulated in the heart during IR injury, demonstrating its implication in the mechanism and therapy of myocardial IR injury (4, 10, 50, 51). Discrepancies in the non-coding RNA signatures observed in IR injury versus acute myocardial infarction are contingent upon the pathological phase, given that these transcripts undergo dynamic fluctuations over time (10, 45).

### 4.1 Oxidative stress and ncRNAs

Oxidative stress emerges when the generation of pro-oxidant molecules outpaces the body's intrinsic neutralizing capabilities (9, 18). This physiological state is primarily driven by the build-up of unstable free radicals, specifically ROS, which can inflict widespread damage on various biological structures and organ systems (9, 18). Research has established a link between such oxidative imbalances and the aberrant regulation of diverse long non-coding RNAs (lncRNAs) and miRNAs (miRNAs) (8, 18, 49–52). While certain non-coding transcripts intensify pathological outcomes, others function as protective agents against ROS-mediated cellular harm. Significantly, current evidence underscores that these regulatory responses often exhibit a high degree of tissue specificity (49–52).

### 4.2 Classification of Non-coding RNAs

The subcellular localization of non-coding RNAs (Table 1) enables discrete roles of the same RNA, by enabling interactions with different functional protein partners and targets of action (53, 54).

**Table 1:** Classification of Noncoding RNAs with cellular localization.

ncRNA type	Primary cellular localization	Reference
miRNAs	Cytoplasm:	(53)
	• microRNA-induced silencing complex (miRISC)	
	• P-bodies (PBs)	
	• Stress granules (SGs)	
circRNAs	Predominantly in the cytoplasm; a subset is also found in the nucleus.	(53)
lncRNAs	Nucleus:	(53, 54)
	• Chromatin	
	• Nucleoplasm	
	• Nuclear speckles	
	• Paraspeckles	
	• Nucleoli	
	Cytoplasm:	
	• Outer mitochondrial membrane	
	• Endoplasmic reticulum (ER) membrane	
	• Cytoskeletal structures	
	• P-bodies (PBs)	
	• Stress granules (SGs)	

#### 4.2.1 MicroRNAs

Among the non-coding RNA species, miRNAs have received the most extensive research attention and are highly prevalent across various heart-related cell lineages, including fibroblasts, endothelial cells, and cardiomyocytes (3, 55–57). These naturally occurring, single-stranded transcripts typically span 18 to 24 nucleotides and primarily reside within the cytoplasm, where they orchestrate numerous metabolic pathways in the circulatory system and significantly influence the progression of myocardial IR damage (45, 53, 56, 57). Evidence suggests that approximately 60% of human messenger RNAs are regulated by miRNAs, with a single miRNA often interacting with over a hundred different mRNA targets (3, 55–60). Specific molecules such as miR-1, miR-133a, and miR-499 are recognized as prominent signalling components concentrated within the heart muscle (45, 55–57). Notably, miR-204 was the inaugural autophagy-associated microRNA identified in cardiac diseases, particularly during IR events (10, 59). To date, 122 distinct miRNAs have been associated with myocardial IR injury, consisting of 56 upregulated and 66 downregulated variants (7).

##### 4.2.1.1. Mechanism of autophagy regulation by miRNAs

**Figure 4:**
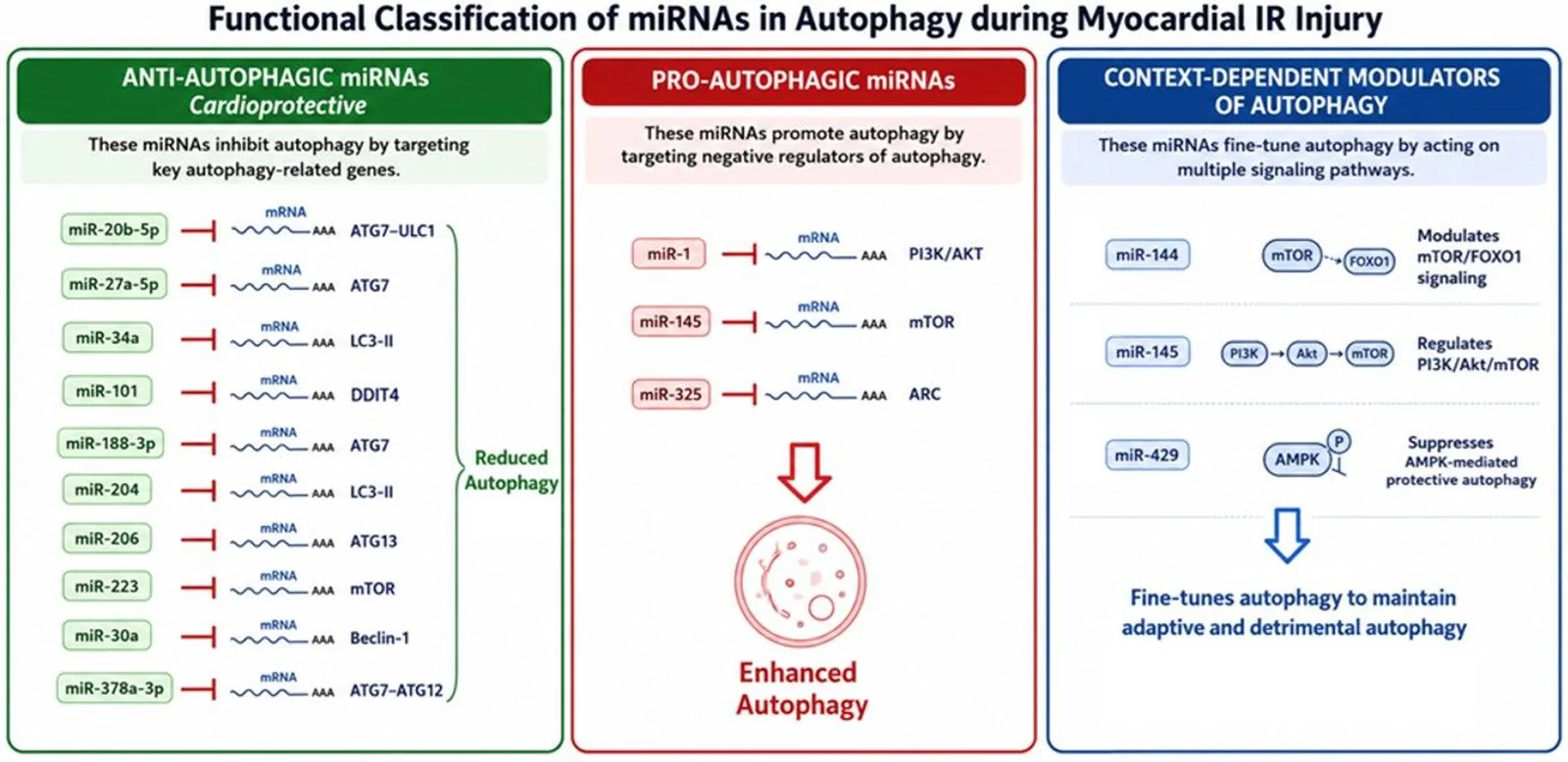
Illustrating functional classification of miRNAs in autophagy during myocardial IR injury.

The miRNAs that regulate autophagy in myocardial IR injury can be broadly divided into three categories based on their biological effects (Table 2) (Figure 4). Anti-autophagic miRNAs constitute the largest group and are predominantly cardioprotective. These include miR-20b-5p, miR-27a-5p, miR-34a, miR-101, miR-188-3p, miR-204, miR-206/216b, miR-223, miR-30a, and miR-378a-3p (58, 59, 62–68, 75), which suppress excessive autophagy through binding to the 3′ Untranslated region (UTR) of mRNAs of autophagy-related genes**,** such as ATG7, Beclin-1, ULK1, ATG13, ATG12, ATG9A, and LC3-II, or by modulating signalling pathways including Akt/mTOR (Table 2). Their inhibition of pathological autophagy reduces cardiomyocyte apoptosis, limits infarct size, and improves myocardial survival during IR. The majority of studies (60%) (miR-20b-5p, miR-27a-5p, miR-204, miR-223, miR-30a, miR-378a-3p) were conducted *in vitro* using hypoxia/reoxygenation-treated cardiomyocytes, while a smaller number of studies (20%) combined cellular and animal models, providing stronger translational evidence for the cardioprotective effects of miR-34a and miR-188-3p. The remaining 20% relied exclusively on animal models, where miR-101 and miR-206/216b were shown to inhibit autophagy and attenuate myocardial IR injury by targeting DDIT4 and ATG13. In contrast, pro-autophagic miRNAs**,** including miR-1 (conducted on animals), while miR-145, and miR-325 (conducted on cells and animals) (70–72, 74)**,** enhance autophagy through pathways such as PI3 K/Akt/mTOR or by targeting ARC. Their effects are context-dependent, as moderate autophagy may facilitate myocardial repair, whereas excessive activation can exacerbate cardiomyocyte injury (70–72, 74). A third group comprises context-dependent modulators**,** including miR-144, (conducted on cells) miR-145 (conducted on animals), and miR-429 (conducted on cells and animals) (69, 71–73)**,** which fine-tune autophagic activity by regulating major signalling pathways such as mTOR, FOXO1, and MO25/LKB1/AMPK. Rather than simply activating or suppressing autophagy, these miRNAs help maintain the balance between adaptive and detrimental autophagy according to the severity and timing of ischemic stress (69, 71–73)**.** Overall, the evidence indicates that most miRNAs exert cardioprotective effects by preventing excessive autophagy, while a smaller subset modulates autophagic flux to preserve myocardial homeostasis and optimize recovery following IR injury.

**Table 2:** Regulatory role of microRNAs in autophagy during myocardial IR injury.

miRNA	Effect on autophagy	Target genes/signalling pathways	Effect on myocardial IR injury	Experimental evidence	Reference
miR-20b-5p	Inhibition	ATG7, ULK1	Inhibits autophagy and decreases H/R-induced injury	Cell	(63)
miR-27a-5p	Inhibition	ATG7	Inhibits autophagy and decreases hypoxia-induced cardiomyocyte injury	Cell	(64)
miR-34a	Inhibition	ATG9A, TNF-alpha	Inhibits autophagy and decreases myocardial IR injury	Cell + Animal	(58)
miR-101	Inhibition	DDIT4	Inhibits autophagy and decreases myocardial infarction-related injury	Animal	(65)
miR-188-3p	Inhibition	ATG7	Inhibits autophagy and decreases myocardial injury	Cell + Animal	(62)
miR-204	Inhibition	LC3-II	Inhibits autophagy and decreases myocardial IR injury	Cell	(59)
miR-206/216b	Inhibition	ATG13	Inhibits autophagy and decreases myocardial IR injury	Animal	(66)
miR-223	Inhibition	PARP-1; Akt/mTOR	Inhibits excessive autophagy and decreases hypoxia-induced injury	Cell	(67)
miR-30a	Inhibition	Beclin-1	Inhibits autophagy and decreases hypoxia-related myocardial injury	Cell	(68)
miR-429	Inhibition	MO25/LKB1/AMPK	miR-429 increases injury; antagonism enhances protective autophagy	Cell	(69)
miR-1	Promotion	PIK3CA; PI3K/AKT	Promotes autophagy; inhibition decreases drug-induced cardiac injury	Animal	(70)
miR-145	Promotion	PI3K/Akt/mTOR; FRS2	Promotes autophagy; context-dependent effects on infarct repair/IR injury	Cell + Animal	(71, 72)
miR-144	Modulation-Inhibits excessive autophagy	mTOR; FOXO1	Limits excessive autophagy and reduces post-MI remodelling	Animal	(73)
miR-325	Promotion	ARC	Promotes autophagy and myocardial injury	Cell + Animal	(74)
miR-378a-3p	Inhibition	ATG12/ATG7 axis	Inhibits autophagy and decreases hypoxia-induced cardiomyocyte injury	Cell	(75)

#### 4.2.2 Long Non-coding RNAs

LncRNAs comprise a category of non-coding transcripts ranging from 200 to 100,000 nucleotides, significantly influencing both health and disease states (10, 39, 42, 50, 75). While a majority of lncRNAs reside within the nucleus, many also operate within the cytoplasmic compartment (53, 54). Their specific subcellular distribution varies across cell types and is dictated by their distinct molecular roles (53, 54). Within the nucleus, these molecules engage with DNA to either repress or stimulate gene transcription. Conversely, those concentrated in the cytoplasm often bind to proteins to fine-tune the translation of mRNA (45, 53, 54). Approximately 40% of the human lncRNA repertoire—representing between 4,000 and 20,000 genes—is uniquely expressed in neural tissues (45, 76). Experimental research using rodent models has demonstrated that myocardial IR injury triggers atypical lncRNA expression patterns in the heart (10, 45, 77). For instance, Liu and colleagues documented 151 lncRNAs with modified expression levels in rat myocardium following IR, comprising 87 downregulated and 64 upregulated species (77). One notable mitochondrial transcript, known as long intergenic non-coding RNA (LIPCAR) predicting cardiac remodelling, exhibits a biphasic expression pattern: it declines during the early stages of myocardial infarction (MI) but increases during later phases (3). Consequently, systemic levels of LIPCAR have been utilized as a diagnostic biomarker for identifying MI and as a predictive indicator for cardiac remodelling and subsequent heart failure (3).

##### 4.2.2.1 Mechanism of autophagy regulation by LncRNAs

**Figure 5:**
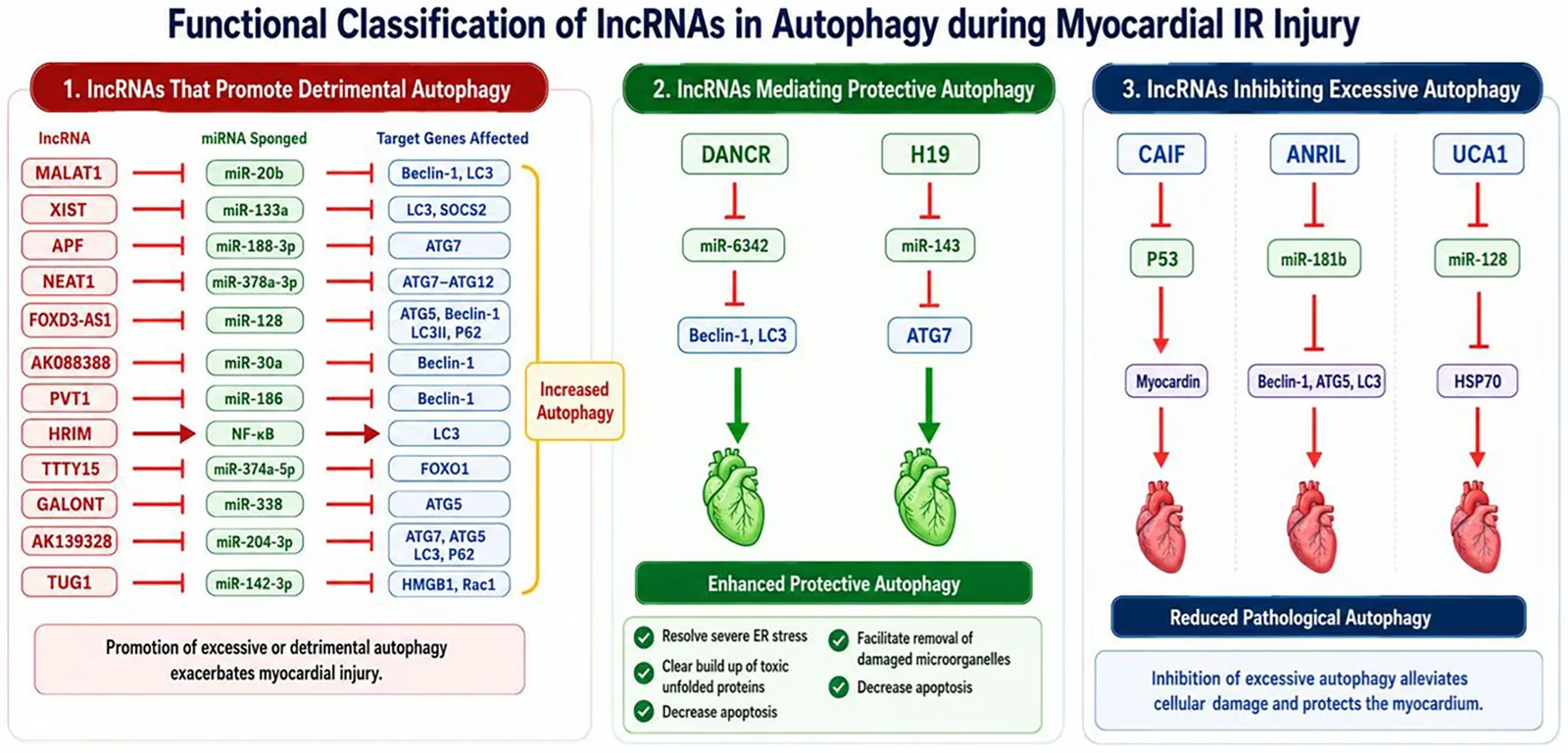
Illustrating functional classification of lncRNAs in autophagy during myocardial IR injury.

Myocardial IR injury is governed by a hierarchical lncRNA-centered regulatory network in which diverse lncRNAs modulate autophagy through different mechanisms (Table 3), (Figure 5).

**Table 3:** Regulatory role of lncRNA s in autophagy during myocardial IR injury.

lncRNA	Expression	Targeted gene/miRNA	Autophagy-related targets	Effect on autophagy	Effect on myocardial IR Injury	Experimental evidence	Reference
MALAT1	Upregulated	miR-20b	Beclin-1, LC3B	Promotion	Promotes OGD/R-induced cardiomyocyte injury	Cell	(85)
H19	Upregulated	miR-143	ATG7, Beclin-1	Promotion	6-Gingerol-associated H19/miR-143/ATG7 signaling activates autophagy and alleviates injury	Cell + Animal	(86)
XIST	Upregulated	miR-133a	LC3, SOCS2	Promotion	XIST inhibition improves injury by suppressing autophagy and regulating SOCS2	Cell + Animal	(50)
APF	Upregulated	miR-188-3p	ATG7	Promotion	Promotes autophagic cell death and increases infarct size	Cell + Animal	(62)
NEAT1	Upregulated	miR-378a-3p	ATG12/ATG7	Promotion	Promotes autophagy and aggravate hypoxia-induced injury	Cell	(75)
FOXD3-AS1	Upregulated	miR-128	ATG5, LC3-II, Beclin-1, p62	Promotion	Reported to aggravate H9c2 H/R injury	Cell	(51)
CAIF	Upregulated	p53	myocardin	Inhibition	Reduces infarct size by inhibiting autophagic cell death	Cell + Animal	(78)
NEAT1 (diabetic I/R)	Upregulated	FOXO1	ATG7, ATG5	Promotion	Promotes autophagy and aggravates diabetic myocardial IR injury	Animal	(82)
AK088388	Upregulated	miR-30a	Beclin-1, LC3	Promotion	Downregulation decreases autophagy and cardiomyocyte death	Cell	(88)
PVT1	Upregulated	miR-186	Beclin-1	Promotion	Knockdown protects AC16 cardiomyocytes from H/R injury	Cell	(80)
HRIM	Upregulated	NF-*κ*B	LC3	Promotion	Inhibition improves myocyte viability during H/R	Cell	(89)
TTTY15	Upregulated	miR-374a-5p	FOXO1	Promotion	Knockdown alleviates MIRI by inhibiting autophagy	Cell + Animal	(90)
ANRIL	Upregulated	niR-181	LC3, Beclin-1	Inhibition	Puerarin-associated ANRIL upregulation inhibits autophagy and protects cardiomyocytes	Cell + Animal	(91)
Galont	Upregulated	miR-338	ATG5	Promotion	Promotes IR-induced autophagy and cardiomyocyte death	Cell	(81)
UCA1	Upregulated	miR-128	HSP70	Inhibition	Reduces myocardial autophagy and apoptosis after IR	Animal	(92)
AK139328	Upregulated	miR-204-3p	ATG7, ATG5, LC3, p62	Promotion	Enhances autophagy within cardiomyocytes	Animal	(93)
DANCR	Upregulated	miR-6324	Beclin-1, LC3	Promotion	Protects cardiomyocytes from ER-stress injury	Cell	(94)
TUG1	Upregulated	miR-142-3p	HMGB1, Rac1	Promotion	Reported to promote autophagy; targeting TUG1 ameliorates injury	Unverified	(79)

###### 4.2.2.1.1 MiRNA Sponging

LncRNAs exert regulatory control over gene expression by sequestering miRNAs, thereby impeding the miRNAs' ability to bind to and modulate their mRNA targets (Figure 5) (3, 35, 77). This mechanism is widely recognized as the “competing endogenous RNA (ceRNA)” hypothesis or the “miRNA sponge” effect (3, 35, 77). Consequently, lncRNAs function as molecular “sponges” for miRNAs, preventing the suppression of downstream target genes (3, 35).

Research highlights several instances of lncRNA involvement in autophagy regulation. For example, Wang et al. demonstrated that lncRNA MALAT1 acts as a sponge for miR-20b (Table 3), leading to an upregulation of Beclin-1 expression and a subsequent increase in autophagic activity (85). Similarly, lncRNAs H19 and XIST (Table 3) participate in autophagy modulation through analogous competitive binding interactions, influencing autophagy pathways via mTOR signalling (35, 50).

Further studies illustrate the diverse roles of lncRNAs (Figure 5). The lncRNA APF (Table 3) has been shown to promote autophagy and impact myocardial infarction by sponging miR-188-3p, which in turn targets ATG7 (3, 10, 62). Independently, Yu et al. corroborated the role of lncRNA AK139328 in enhancing autophagy within cardiomyocytes by inhibiting miR-204-3p (93). Another lncRNA, NEAT1 (Table 3), contributes to autophagy promotion by sponging miR-378a-3p and modulating ATG12 along with other related autophagic factors (75). Investigations by Li et al. into lncRNA XIST in myocardial IR injury revealed an elevation in its levels following injury and promotion of autophagy through inhibition of miR-133a (50). Inhibition of XIST expression was observed to mitigate cellular damage and improving myocardial IR injury suggesting its potential as a therapeutic target for myocardial IR injury (50, 77).

FOXD3-AS1 is upregulated in cardiomyocytes under IR conditions (Figure 5). It acts as a ceRNA by sponging miR-135a-5p, reducing its availability (35, 51, 98). miR-135a-5p normally suppresses the expression of ATG7, a gene crucial for autophagy. When miR-135a-5p is inhibited by FOXD3-AS1, ATG7 expression increases**,** enhancing autophagy. Overexpression of ATG7 results in increased autophagic activity**,** which, in this context, leads to cell damage during reperfusion phase of IR injury (35, 51, 98). Silencing FOXD3-AS1 - Increases miR-135a-5p - Reduces ATG7 - Decreases harmful autophagy, improves cardiomyocyte survival during reperfusion phase and opens the door for therapeutic potentials (35, 51, 98).

###### 4.2.2.1.2 Transcriptional Control

CAIF (Table 3) directly binds to p53, preventing it from activating transcription of myocardin and regulates autophagy independently of miRNAs (Figure 5) (78).

###### 4.2.2.1.3 Signalling Pathways

TUG (Table 3) (Figure 5) Identifies multi-target downstream pathway (HMGB1 + Rac1). TUG can regulate entire signalling cascades, not just single genes (79). The function of TUG1 is achieved by sponging miR142-3p leading to the release of suppression of the putative targets of miR-142-3p, HMGB1 and Rac1. Both HMGB1 and Rac1 essentially mediate cell apoptosis and autophagy. So TUG1 sponging of miR142-3p will promote autophagy and apoptosis; and worsen myocardial IR injury. Inhibition of TUG1 ameliorates myocardial IR injury (79).

All these pathways (Table 3) (Figure 5) converge on core autophagy machinery (multiple autophagy proteins - Beclin1, ATG5, ATG7) (62, 80, 81) and autophagic flux (dysregulated autophagic flux- NEAT1) (82). Research on lncRNAs is challenging because, unlike miRNAs, most lncRNAs are not conserved across species (83). Additionally, the inadequate yield of high-quality RNA from the peripheral blood restricts the use of lncRNAs as clinical biomarkers (84).

#### 4.2.3 Circular RNAs

CircRNAs represent a distinct category of ncRNAs characterized by their covalently closed loop structure, which distinguishes them from linear RNA molecules (41, 55, 95–97). These molecules are increasingly recognized as significant biomarkers across a spectrum of biological processes (41, 55, 97). Predominantly localized within the cytoplasm (Table 4) (41, 53), circRNAs exhibit enhanced biological stability compared to their linear counterparts (41, 55). This inherent stability, conferred by their unique closed conformation, enables them to play crucial roles, particularly in the context of myocardial IR injury (97).

**Table 4:** Regulatory role of circRNAs in autophagy during myocardial IR injury.

circRNA	Expression	Targeted miRNA	Autophagy-related targets	Effect on autophagy	Effect on myocardial IR Injury	Experimental evidence	Reference
circPAN3	Downregulated	miR-421	PINK1	Inhibition	Upregulation leads to suppression of autophagy and improves myocardial IR injury	Cell + Animal	(95)
ACR	Upregulated	-	PINK1; FAM65B	Inhibition	Attenuates IR injury by suppressing autophagy and cell death	Cell + Animal	(8)
circHIPK3	Upregulated	miR-20b-5p	ATG7, Beclin-1	Promotion	Promotes autophagy/apoptosis and aggravates H/R-induced cardiomyocyte injury	Cell	(87)
hsa_circ_0000064	Not determined	-	p62, LC3, Beclin-1	Inhibition	Salidroside upregulates hsa_circ_0000064 leading to suppression of autophagy and improves IR injury	Cell + Animal	(96)

Despite their growing recognition, the functional understanding of circRNAs remains less developed compared to miRNAs and lncRNAs (41, 45). Nevertheless, specific circRNAs, such as circSLC8A1, circCACNA1D, circSPHKAP, and circALPK2, are found in high abundance within cardiomyocytes and hold promise as circulating biomarkers detectable in blood (41, 53, 55, 97, 98). Emerging research has begun to elucidate the involvement of several circRNAs, including circPAN3, circ-TLK1, circ-ACR, circ-100338, and circ-0068566, in the pathophysiology of myocardial IR injury (7, 10, 98).

One notable example is circPAN3, which has been observed to be downregulated during myocardial IR injury. Experimental overexpression of circPAN3 has been shown to mitigate autophagy, evidenced by a reduction in autophagic vacuoles. This protective effect of circPAN3 on the heart is mediated through the circPAN3–miR-421–Pink1 signalling pathway, which critically regulates autophagic processes (95, 98).

##### 4.2.3.1 Mechanism of autophagy regulation by CircRNAs

**Figure 6:**
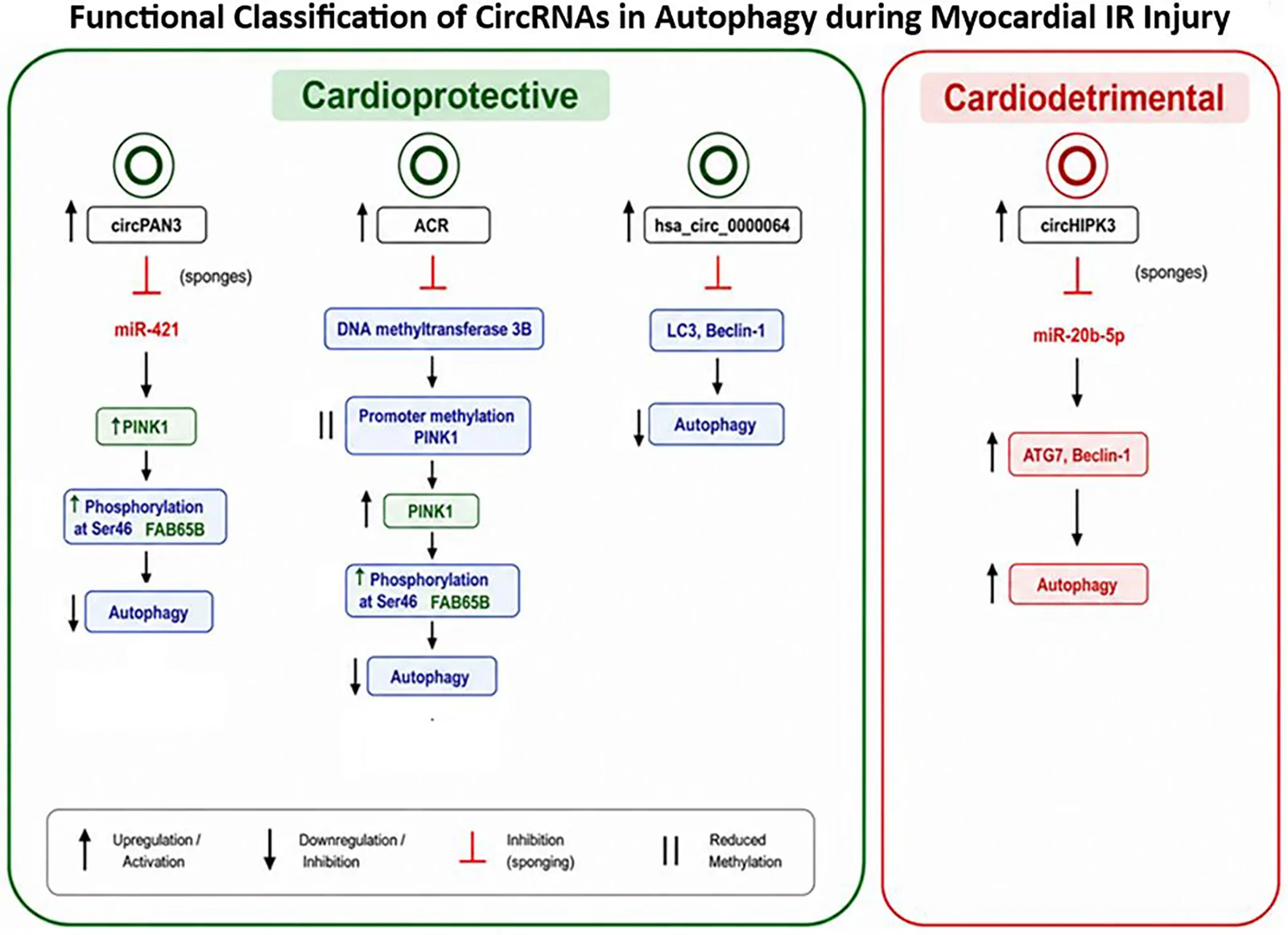
Illustrating functional classification of circRNAs in autophagy during IR injury.

Recent studies have also shown a role of circRNAs as ceRNAs that sponge specific miRNAs and prevent them from suppressing their target mRNAs (Table 4) (Figure 6) (2, 35, 97). This allows for increased expression of autophagy-related proteins, influencing cell survival or death during IR injury (97). circPAN3 and circHIPK3 regulate myocardial IR injury primarily via miRNA sponging, whereas ACR regulates gene transcription epigenetically by DNA methylation mechanisms (2, 8, 35, 97). Gong et al. highlight that the lncRNA/circRNA–miRNA–mRNA axes are crucial regulators of autophagy during IR injury (97). These pathways represent potential therapeutic targets for protecting the heart by restoring autophagy balance (Figure 4).

Autophagy-related circular RNA (ACR) plays a crucial role in regulating cardiomyocyte autophagy by directly interacting with DNA methyltransferase 3B (Dnmt3B) (2, 8, 97, 98). This interaction prevents Dnmt3B from methylating the promoter region of Pink1, thereby enhancing Pink1 expression (2, 8, 97, 98). Subsequently, Pink1 phosphorylates FAM65B at serine 46, which leads to the inhibition of autophagy and a reduction in myocardial infarction size (2, 8, 97, 98). These findings collectively highlight the protective function of the ACR/Pink1/FAM65B axis in the context of myocardial IR injury (2, 8, 97, 98). Song et al. describe circRNAs like circHIPK3 (Table 4) functioning in competing endogenous RNA **(**ceRNA) networks, affecting the PI3 K/Akt/mTOR pathway, a central regulator of autophagy (61, 95).

Another significant circRNA, circPAN3, is observed to be downregulated during myocardial IR injury (53, 95). Its overexpression has been shown to markedly suppress autophagy, as evidenced by a decrease in autophagic vacuoles (35, 95, 97, 98). Furthermore, circPAN3 directly targets miR-421, which in turn negatively regulates Pink1 through specific binding sites, thereby influencing myocardial IR injury (35, 95, 97, 98). The abrogation of circPAN3-induced anti-autophagy effects by Pink1 downregulation underscores the critical involvement of the circPAN3-miR-421-Pink1 signalling pathway in mediating autophagy and conferring cardioprotection during IR injury (35, 95, 97, 98).

### 4.3 Strength of evidence supporting the role of ncRNAs in regulating autophagy during myocardial IR injury

The strength of evidence supporting the role of ncRNAs in regulating autophagy during myocardial IR injury varies considerably across the studies summarized in Tables 2–4. The most convincing evidence comes from ncRNAs that have been independently validated in both *in vitro* and *in vivo* models, demonstrating consistent modulation of autophagy and corresponding effects on myocardial injury. Among miRNAs, miR-34a, miR-145, miR-188-3p, miR-325, and miR-144 have relatively strong experimental support, as their roles have been investigated in cultured cardiomyocytes together with rodent models of AMI or IR injury (58, 62, 71–74, 73).

Similarly, among lncRNAs; APF, H19, XIST, ANRIL, TTTY15, and AK139328 are supported by animal studies in addition to mechanistic cellular experiments, providing stronger evidence for their involvement in autophagy-mediated cardioprotection or injury (50, 62, 86, 90, 91, 93). The circular RNAs ACR and circPAN3 also have comparatively robust evidence because their effects have been demonstrated in both cultured cardiomyocytes and murine IR models (7, 8, 95). Furthermore, the APF/miR-188-3p regulator*y* axis and the Pink1-regulating circRNA ACR have been investigated by multiple independent studies and have become among the most frequently cited ncRNA-mediated autophagy pathways in MIRI, increasing confidence that these represent biologically relevant regulatory mechanisms rather than isolated observations (8, 62).

In contrast, many ncRNAs—including miR-20b-5p, miR-27a-5p, miR-204, miR-223, miR-429, miR-378a-3p, MALAT1, NEAT1, AK088388, PVT1, HRIM, Galont, Dancr, TUG1, circHIPK3, and circ_0000064—remain supported primarily by single studies using cell culture models or limited experimental systems (59, 63, 64, 67, 69, 79–81, 85, 87–89, 94, 96). Although these findings provide valuable mechanistic insights, they require independent validation in additional animal models and, ultimately, in human myocardial tissues before their biological significance and therapeutic potential can be firmly established.

Importantly, none of the ncRNAs summarized in these tables have been validated in large human myocardial tissue cohorts or prospective clinical studies. Only a few investigations employed human-derived cell lines, such as AC16 cardiomyocytes in studies of PVT1, which, although useful for mechanistic investigation, do not represent clinical validation (80). Therefore, despite the growing body of experimental evidence implicating ncRNAs in autophagy regulation during myocardial IR injury, the field remains largely preclinical, and the translational potential of many promising ncRNA candidates awaits confirmation in well-designed human studies

## 5 Regulation of autophagy by ncRNAs at different stages of autophagy

**Figure 7:**
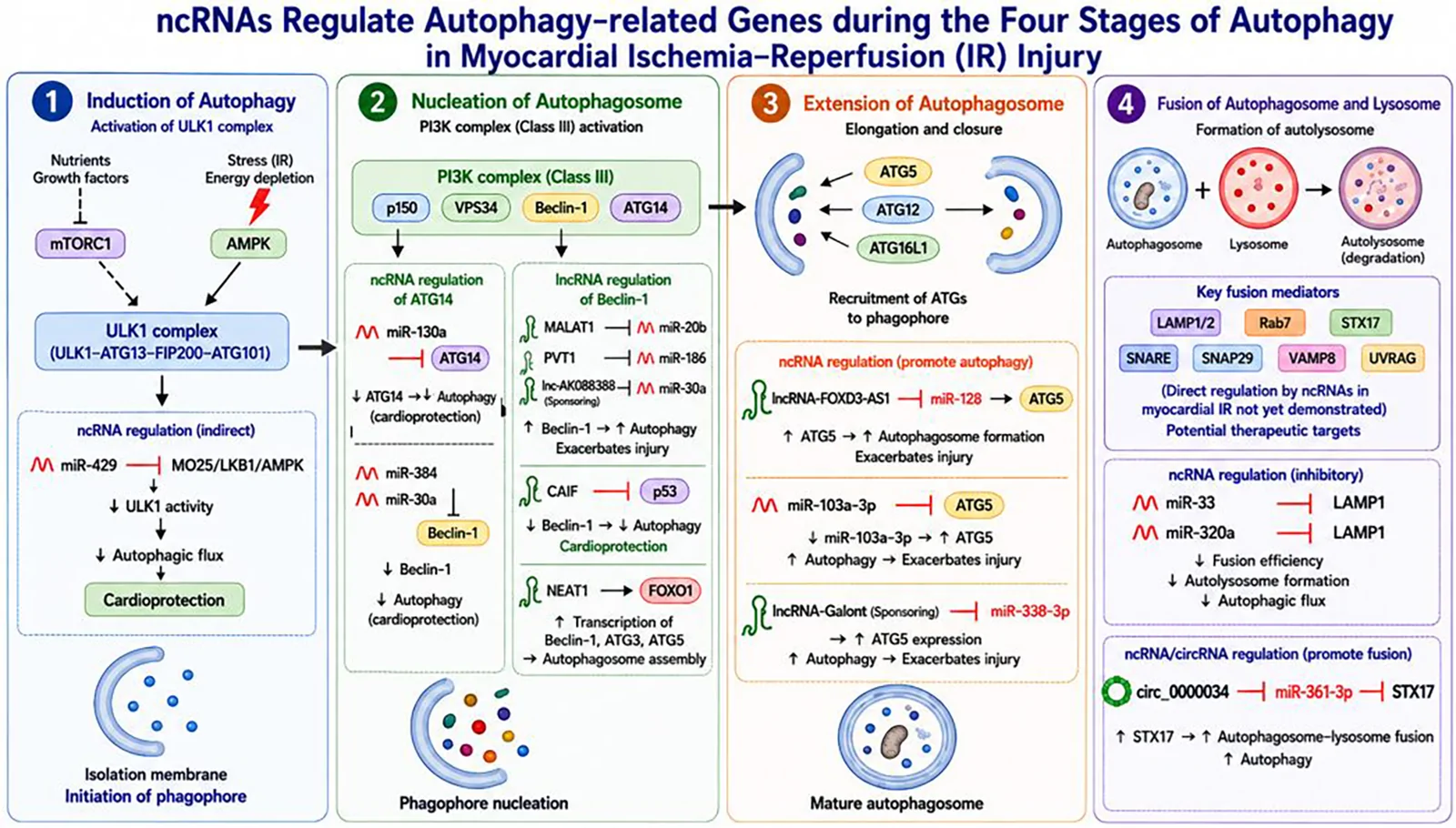
Illustrating the regulation of autophagy-related-proteins by ncRNAs during myocardial IR injury.

The ncRNAs affect the outcome of myocardial IR injury by regulating different stages of autophagy; induction, nucleation of autophagosome, formation and extension autophagosome, and the fusion of autophagosome with lysosome (Figure 7) (35, 98).

### 5.1 Induction of autophagy

Autophagy is initiated through activation of the ULK1/2 kinase complex, consisting of ULK1/2, ATG13, FIP200, and ATG101, which integrates upstream nutrient- and energy-sensing signals from mTORC1 and AMPK (35, 98). Under nutrient-rich conditions, mTORC1 phosphorylates ULK1 and ATG13, maintaining the complex in an inactive state (35, 98). During ischemic stress, inhibition of mTORC1 together with AMPK activation releases this suppression, allowing ULK1 activation and phosphorylation of downstream autophagy proteins, including Beclin-1 and VPS34, thereby initiating autophagosome formation (35, 98). Among ncRNAs, miR-429 has emerged as an important indirect regulator of this stage (69). Zhu Q et al. demonstrated that miR-429 indirectly directly targeted ULK1 complex. During myocardial IR injury, miR-429 functions as an upstream regulator of autophagy initiation by modulating the MO25 (CAB39)/LKB1/AMPK signalling pathway. miR-429 directly targets MO25, a critical activator of the LKB1–AMPK axis, thereby suppressing AMPK activation leading to inhibition of ULK1–ATG13 autophagy initiation complex. Inhibition of miR-429 restores MO25/LKB1/AMPK signalling, resulting in increased activation of the ULK1–ATG13 autophagy initiation complex and enhanced autophagic flux, highlighting miR-429 as an indirect regulator of autophagy initiation rather than a direct regulator of ULK1 (Figure 7) (69).

### 5.2 Nucleation of the autophagosome

Following ULK1 activation, nucleation of the phagophore depends on the class III phosphatidylinositol-3 kinase (PI3KC3) complex, which consists of VPS34, VPS15 (p150), Beclin-1, and ATG14. This complex generates phosphatidylinositol-3-phosphate (PI3P), recruiting WIPI proteins and additional ATG proteins required for phagophore formation (35, 98, 99). Within this complex, ATG14 functions as a key organizer by targeting the PI3KC3 complex to the isolation membrane while enhancing VPS34 activity (35, 98–100). miR-130a directly inhibits ATG14; therefore, inhibition of miR-130a increases ATG14 expression, enhances Beclin-1 phosphorylation, and promotes autophagic flux (Figure 7) (100).

Beclin-1 is the central regulator of autophagosome nucleation and is highly expressed during reperfusion (10). Increased Beclin-1 promotes excessive autophagy and contributes to myocardial injury, whereas suppression of Beclin-1 generally attenuates reperfusion-induced damage (6, 10, 101, 102). Accordingly, miR-30a and miR-384 directly target Beclin-1, limiting excessive autophagy and conferring cardioprotection (Figure 7) (102, 103).

Several lncRNAs regulate Beclin-1 through competitive endogenous RNA (ceRNA) mechanisms. MALAT1, PVT1, and AK088388 sponge miR-20b, miR-186, and miR-30a, respectively (Figure 7), thereby relieving repression of Beclin-1 and enhancing autophagy, which exacerbates myocardial IR injury (35, 98). Interestingly, p53 functions as a transcriptional activator by binding directly to the promoter region of the myocardin gene, upregulating its mRNA and protein expression levels (35, 98). Elevated myocardin expression downstream of p53 leads to the activation of Beclin-1, which initiates autophagy and can trigger autophagic cell death in cardiomyocytes during myocardial IR injury (35, 78, 98). The lncRNA CAIF binds directly to the p53 protein to block p53-mediated myocardin transcription, thereby protecting heart tissue from excessive autophagy and ischemic damage (35, 78, 98). Therefore, loss of CAIF enhances Beclin-1 expression, increases autophagy, and aggravates myocardial injury. In addition, NEAT1 promotes autophagosome formation through epigenetic activation of FOXO1, resulting in increased transcription of BECN1, ATG3, and ATG5, thereby facilitating both nucleation and subsequent autophagosome expansion (Figure 7) (104)**.**

### 5.3 The extension of autophagosome

Autophagosome elongation is mediated by two ubiquitin-like conjugation systems: the ATG12–ATG5–ATG16L1 complex and the LC3/ATG8 lipidation pathway. These systems coordinate membrane expansion and closure of the growing autophagosome (51, 81, 105).

Within the ATG12–ATG5–ATG16L1 pathway, several ncRNAs regulate ATG5, a pivotal scaffold protein required for membrane elongation. FOXD3-AS1 enhances ATG5 expression and promotes autophagy, aggravating myocardial ischemia–reperfusion injury (35, 51, 98). Similarly, lncRNA Galont functions as a ceRNA by sponging miR-338, thereby increasing ATG5 expression and stimulating autophagosome formation (35, 81). While miR-103a-3p has been experimentally shown to inhibit autophagy by decreasing ATG5 expression and reducing cardiomyocyte injury during IR, although whether this regulation is direct or occurs through inhibition of upstream repressors remains to be fully elucidated (98, 104). Therefore, miR-103a-3p should be considered a negative regulator of ATG5-dependent autophagosome elongation (Figure 7) (98, 104).

Additional ncRNAs regulate other components of the elongation machinery. NEAT1 and AK139328 increase ATG5 and ATG7 expression, particularly in diabetic myocardial IR injury models, thereby enhancing autophagy (82, 93). lncRNA APF and circHIPK3 modulate ATG7 through miR-188-3p and miR-20b-5p, respectively (5, 62), whereas H19 promotes ATG7 expression by sponging miR-143 (Figure 7) (39). Collectively, these ncRNAs fine-tune autophagosome membrane expansion and maturation by regulating components of both ubiquitin-like conjugation systems.

### 5.4 Fusion of autophagosome and lysosome

Autophagosome–lysosome fusion represents the terminal stage of autophagic flux, during which mature autophagosomes fuse with lysosomes to form autolysosomes for degradation of damaged proteins and dysfunctional organelles. This process is coordinated by several fusion-associated proteins, including lysosome-associated membrane protein-1/2 (LAMP1/2), Ras-related in brain protein (Rab7), and the Soluble N-ethylmaleimide-sensitive factor Attachment Protein Receptor (SNARE) complex composed of syntaxin-17 (STX17), Synaptosome-Associated Protein 29 (SNAP29), ultraviolet radiation resistance-associated gene protein

(UVRAG), and vesicle-associated membrane protein 7/8 (VAMP7/8), which collectively ensure efficient autophagic cargo clearance (5, 106, 109). In the heart, disruption of autophagosome–lysosome fusion results in impaired autophagic flux, accumulation of undegraded autophagosomes, mitochondrial dysfunction, and enhanced cardiomyocyte death during myocardial IR injury (35, 108, 110). Although numerous studies have demonstrated that miRNAs, lncRNAs, and circRNAs regulate autophagy initiation and autophagosome formation in myocardial IR injury, no original experimental study has yet demonstrated direct regulation of autophagosome–lysosome fusion by ncRNAs in this disease (111). Few studies on non-cardiac tissue revealed several miRNAs, specifically miR-320a, miR-33, act as inhibitory regulators of this autophagic stage (35, 112–114). These miRNAs exert their suppressive effects by targeting and downregulating essential fusion mediators like LAMP1, thereby impeding the maturation of autolysosomes, (Figure 7) (112–114). In addition, circ_0000034 sponges miR-361-3p leading to removing the suppression of STX17. The upregulation of STX17 will then leads to increased phagolysosome fusion and increase autophagy (Figure 7) (35).

Consequently, the potential involvement of ncRNAs in regulating fusion-associated proteins such as LAMP1/2, STX17, Rab7, or the SNARE complex during myocardial IR injury remains speculative. Elucidating whether ncRNAs modulate the late stages of autophagic flux represents an important area for future research and may identify novel therapeutic targets for restoring lysosomal function and improving cardiomyocyte survival following IR injury.

### 5.5 Temporal dynamics of ncRNA-mediated autophagy during myocardial IR injury

The expression and biological effects of ncRNAs are highly dynamic throughout myocardial IR injury, reflecting the changing physiological requirements of cardiomyocytes during ischemia and reperfusion (35, 98). During the ischemic phase, energy depletion, hypoxia, and AMPK activation generally favor the induction of adaptive autophagy, which maintains cellular homeostasis by recycling damaged proteins and organelles and preserving ATP production (35, 98). Accordingly, ncRNAs that enhance ULK1 activation, Beclin-1 signalling, or ATG protein expression may promote cardioprotective autophagy during this stage (35, 98). In contrast, the reperfusion phase is characterized by abrupt restoration of oxygen supply, excessive reactive oxygen species generation, calcium overload, mitochondrial dysfunction, and inflammatory activation, conditions that frequently lead to excessive or dysregulated autophagy (35, 98). At this stage, altered expression of several miRNAs and lncRNAs—including those targeting Beclin-1, ATG5, and ATG7—can amplify autophagic activity or impair autophagic flux, thereby promoting cardiomyocyte death rather than survival (35, 98). For example, miR-30a directly targets the 3′-untranslated region of BECN1, suppressing Beclin-1 expression and limiting excessive autophagy during reperfusion, thereby reducing myocardial IR injury (35, 98, 102, 103). However, because Beclin-1 also participates in autophagic flux regulation, conflicting findings have been reported, with some studies suggesting that miR-30a downregulation enhances protective autophagy under certain experimental conditions (35, 98, 102, 103). These temporal and context-dependent effects may explain the apparently contradictory roles reported for miR-30a and other Beclin-1-regulating ncRNAs (98, 103). Therefore, the biological function of a given ncRNA should always be interpreted within the temporal context of ischemia versus reperfusion, emphasizing that therapeutic strategies should aim to restore physiological autophagic balance in a stage-specific manner rather than uniformly enhancing or suppressing autophagy (35, 98, 102, 103).

## 6 Clinical translation of autophagy-related ncRNAs in myocardial IR injury

Despite substantial progress in defining the roles of ncRNAs in myocardial IR injury, their clinical translation remains limited. Among the three principal ncRNA classes, miRNAs currently have the strongest human evidence. Circulating miRNAs—including miR-1, miR-133a, miR-145, miR-499, miR-223 and related cardiac-enriched miRNAs (Table 5) have been evaluated in patients with AMI and acute coronary syndromes for diagnosis, risk stratification and prediction of cardiovascular outcomes, including in patients treated with primary percutaneous coronary intervention (PCI) (115–125). However, their diagnostic and prognostic performance is heterogeneous, and none has yet demonstrated sufficient incremental value over high-sensitivity cardiac troponin, natriuretic peptides, electrocardiography and cardiac imaging to support routine clinical implementation (122, 124).

**Table 5:** Clinical translation landscape of autophagy-related miRNAs during MI and coronary reperfusion.

ncRNA	Direction/specimen	Clinical domain	Evidence in MI with PCI/reperfusion	Key human or mechanistic finding	Translation stage	Key refs.
miR-1	Usually increased; plasma/serum	Diagnosis; injury burden; prognosis	Measured miR-1 in plasma PCI cohorts; correlated positively with NT-proBNP and hsTnT 24 h after admission and negatively with left ventricular ejection fraction (LVEF)	PRAGUE-18 substudy: miR-1 correlated with conventional injury and functional markers after AMI treated with plasma PCI. Earlier studies support diagnostic signal, with heterogeneity in timing and normalization.	Human investigational	(115–117)
miR-133a	Usually increased; plasma/serum	Diagnosis; injury burden; prognosis	Measured miR-133a in plasma PCI cohorts; correlated positively with NT-proBNP and hsTnT 24 h after admission and negatively with left ventricular ejection fraction (LVEF)	PRAGUE-18 substudy: miR-133a correlated with conventional injury and functional markers after AMI treated with plasma PCI. Earlier studies support diagnostic signal, with heterogeneity in timing and normalization.	Human investigational	(115–117)
miR-499	Usually increased; plasma/serum	Diagnosis; injury burden; prognosis	Measured miR-499 in plasma PCI cohorts; correlated positively with NT-proBNP and hsTnT 24 h after admission and negatively with left ventricular ejection fraction (LVEF)	PRAGUE-18 substudy: miR-499 correlated with conventional injury and functional markers after AMI treated with plasma PCI. Earlier studies support diagnostic signal, with heterogeneity in timing and normalization. Plasma levels of circulating miR-499 could contribute to the identification of patients at increased risk of death during the first year after AMI, especially when combined with NT-proBNP levels.	Human investigational	(115–117)
miR-223	Variable; plasma/platelet-related	Prognosis; platelet/inflammatory risk	Human CAD/ACS prognostic data exist, but direct validation as a reperfusion-monitoring test is insufficient.	Circulating miR-223 has been associated with cardiovascular death in symptomatic CAD; mechanistically it modulates autophagy through PARP-1/Akt-mTOR in cardiomyocytes.	Early translational	(67, 118)
miR-145	Usually decreased in CAD	CAD severity; vascular phenotype	Coronary angiography cohorts link lower levels with CAD severity and STEMI; not validated for post-PCI follow-up.	Lower plasma miR-145 was associated with multivessel disease, higher SYNTAX score and STEMI in patients undergoing angiography.	Human investigational	(71, 72, 119)
miR-144	Variable	Remodelling/repair	No validated human reperfusion-monitoring study identified.	Evidence in the autophagy-focused source is animal based, including reduced LV remodelling after experimental MI.	Preclinical	(73)
miR-30a	Variable	Potential diagnostic/remodelling signal	No validated PCI-specific clinical role.	Mechanistic evidence supports exosomal transfer and regulation of Beclin-1-dependent autophagy after hypoxia.	Preclinical	(68)
miR-20b-5p	Not established clinically	None established	No patient validation.	Cell evidence: inhibition of ATG7/ULK1-related autophagy in H/R-stimulated endothelial cells.	Preclinical	(63)
miR-27a-5p	Not established clinically	None established	No patient validation.	Cell evidence: targets ATG7 and attenuates hypoxia-induced cardiomyocyte injury.	Preclinical	(64)
miR-101	Not established clinically	None established	No patient validation.	Animal evidence: targets DDIT4 and modulates autophagy after MI.	Preclinical	(65)
miR-188-3p	Not established clinically	None established	No patient validation.	Cell/animal evidence within the APF-miR-188-3p-ATG7 axis.	Preclinical	(62)
miR-204	Not established clinically	None established	No patient validation.	Cell evidence: regulates ischemia/reperfusion-induced cardiomyocyte autophagy through LC3-II.	Preclinical	(59)
miR-206/216b	Not established clinically	None established	No patient validation.	Animal evidence: ATG13-dependent autophagic apoptosis in histamine-deficient cardiac injury.	Preclinical	(66)
miR-429	Not established clinically	None established	No patient validation.	Cell evidence: antagonism enhances MO25/LKB1/AMPK-mediated protective autophagy.	Preclinical	(69)
miR-325	Not established clinically	None established	No patient validation.	Cell/animal evidence: promotes an autophagic program and myocardial injury.	Preclinical	(74)
miR-378a-3p	Not established clinically	None established	No patient validation.	Cell evidence within the NEAT1-miR-378a-3p-ATG12/ATG7 axis.	Preclinical	(76)

Several autophagy-related miRNAs included in the translational table (Table 5) have promising mechanistic relevance but remain predominantly preclinical. miR-223, miR-34a, miR-145 and miR-144 regulate cardiomyocyte autophagy, apoptosis, infarct repair and ventricular remodelling in experimental models (58, 67, 71–73), whereas miR-20b-5p, miR-27a-5p, miR-101, miR-188-3p, miR-204, miR-206/216b, miR-30a, miR-429, miR-325 and miR-378a-3p have been studied mainly in cellular or animal models of hypoxia/reoxygenation, myocardial infarction or IR injury (62–66, 68, 69, 74, 76, 85). Accordingly, these molecules should presently be regarded as mechanistic candidates rather than clinically validated biomarkers.

The translational evidence for lncRNAs is less mature. Among the lncRNAs summarized, circulating exosomal MALAT1 (Table 6) has been evaluated in patients with acute myocardial infarction, including assessment of diagnostic performance and prognosis after coronary stent implantation (120). Plasma H19 (Table 6) has also shown preliminary diagnostic potential in myocardial infarction (121). Nevertheless, most autophagy-related lncRNAs; including MALAT1 in mechanistic studies, H19, NEAT1, ANRIL, UCA1, XIST, CAIF, FOXD3-AS1, AK088388, PVT1, HRIM, TTTY15, Galont, AK139328, DANCR and TUG1—remain supported mainly by cell or animal experiments demonstrating regulation of Beclin-1, ATG proteins, LC3, FOXO1 and related autophagy pathways (50, 51, 75, 78–82, 85, 86, 88–94). Therefore, only MALAT1 and H19 currently have direct human biomarker evidence within this reference set, and neither is used routinely in clinical practice.

**Table 6:** Clinical translation landscape of autophagy-related lncRNAs during MI and coronary reperfusion.

RNA	Direction/specimen	Clinical domain	Evidence in MI with PCI/reperfusion	Key human or mechanistic finding	Translation stage	Key refs.
MALAT1	Usually increased; plasma exosomes	Diagnosis; possible follow-up	Direct evidence after stent implantation: AMI vs. normal-coronary controls with 1-year MACE ascertainment.	Exosomal MALAT1 showed modest discrimination for AMI (AUC 0.707); the study did not establish independent prognostic utility for MALAT1.	Human investigational	(85, 120)
H19	Increased; plasma	Diagnosis	Human MI case-control evidence; PCI-specific serial validation not established.	A small study reported higher plasma H19 in MI and 70% sensitivity/94% specificity at a selected threshold; external validation is lacking.	Human investigational	(86, 121)
NEAT1	Usually increased	Inflammation/prognosis hypothesis	No validated reperfused-MI biomarker role.	The supplied evidence is cell and diabetic-rat IR work linking NEAT1 to ATG12/ATG7 or FOXO1-associated autophagy.	Preclinical	(75, 82)
ANRIL	Variable	CAD susceptibility/severity hypothesis	No validated serial post-PCI role.	The supplied autophagy evidence is experimental puerarin-associated protection, not a clinical biomarker study.	Early translational	(91)
UCA1	Variable	Potential diagnostic hypothesis	No validated PCI evidence.	The supplied evidence is an animal morphine-postconditioning model showing reduced autophagy and apoptosis.	Preclinical	(92)
XIST	Increased experimentally	None established	No patient validation.	Cell/animal evidence: XIST inhibition improved IR injury through miR-133a/SOCS2 and autophagy suppression.	Preclinical	(50)
APF	Increased experimentally	None established	No patient validation.	Cell/animal evidence: APF promotes autophagic cell death through miR-188-3p/ATG7.	Preclinical	(62)
CAIF	Increased experimentally	None established	No patient validation.	Cell/animal evidence: blocks p53-mediated myocardin transcription and autophagic cell death.	Preclinical	(78)
FOXD3-AS1	Increased experimentally	None established	No patient validation.	Cell evidence: promotes ATG5/LC3-II/Beclin-1-associated autophagy and H/R injury.	Preclinical	(51)
AK088388	Increased experimentally	None established	No patient validation.	Cell evidence: regulates miR-30a/Beclin-1/LC3-mediated autophagy.	Preclinical	(88)
PVT1	Increased experimentally	None established	No patient validation.	AC16-cell evidence: knockdown protects through miR-186/Beclin-1.	Preclinical	(80)
HRIM	Increased experimentally	None established	No patient validation.	Cell evidence: inhibition improves myocyte viability by modulating autophagy during H/R.	Preclinical	(89)
TTTY15	Increased experimentally	None established	No patient validation.	Cell/animal evidence: knockdown protects through miR-374a-5p/FOXO1.	Preclinical	(90)
Galont	Increased experimentally	None established	No patient validation.	Cell evidence: miR-338/ATG5-associated autophagy and cardiomyocyte death.	Preclinical	(81)
AK139328	Increased experimentally	None established	No patient validation.	Diabetic-mouse evidence: silencing protects through miR-204-3p and autophagy inhibition.	Preclinical	(93)
DANCR	Increased experimentally	None established	No patient validation.	Cell evidence in ER-stress injury; relevance to clinical reperfused MI remains uncertain.	Preclinical	(94)
TUG1	Increased experimentally	None established	No reliable clinical validation.	The supplied table labels the supporting myocardial IR evidence as unverified; use cautiously.	Preclinical	(79)

Clinical development of circRNAs is even more limited. circPAN3, ACR, circHIPK3 and hsa_circ_0000064 (Table 7) have demonstrated cardioprotective or detrimental effects through regulation of PINK1-mediated mitophagy, FAM65B, ATG7, Beclin-1 and related autophagic pathways (8, 87, 95, 96). However, all four remain preclinical, and none has been prospectively validated for diagnosing myocardial infarction, assessing reperfusion success, predicting post-PCI remodeling or monitoring patients during follow-up.

**Table 7:** Clinical translation landscape of autophagy-related circRNAs during MI and coronary reperfusion.

RNA	Direction/specimen	Clinical domain	Evidence in MI with PCI/reperfusion	Key human or mechanistic finding	Translation stage	Key refs.
circPAN3	Increased experimentally	None established	No human MI/PCI validation identified.	Cell/animal evidence: circPAN3 sponges miR-421, preserves PINK1 signaling and suppresses autophagy in myocardial IR.	Preclinical	(95)
ACR	Increased experimentally	None established	No human MI/PCI validation identified.	Cell/animal evidence: ACR limits autophagic cell death via PINK1/FAM65B signaling.	Preclinical	(8)
circHIPK3	Increased experimentally	None established	No human MI/PCI validation identified.	Cell evidence: circHIPK3/miR-20b-5p/ATG7 axis promotes autophagy and apoptosis after H/R.	Preclinical	(87)
hsa_circ_0000064	Increased experimentally	None established	No human MI/PCI validation identified.	Cell/animal evidence: salidroside-associated regulation of p62, LC3 and Beclin-1 reduced apoptosis/autophagy in IR models.	Preclinical	(96)

Overall, the current evidence demonstrates a clear translational hierarchy: miRNAs have the most extensive human biomarker data, selected lncRNAs—particularly MALAT1 and H19—have early clinical evidence, whereas circRNAs remain almost entirely experimental (115–125). No autophagy-related ncRNA is currently recommended in the 2023 ESC acute coronary syndrome guidelines for diagnosis, prognostic assessment, evaluation of reperfusion efficacy or follow-up after PCI (122). Future studies should prioritize prospective multicentre cohorts, standardized blood-processing and normalization methods, serial sampling before and after reperfusion, and evaluation of whether ncRNA panels provide incremental value beyond established biomarkers and imaging modalities.

In summary, circulating miRNAs represent the most clinically advanced ncRNA class, whereas MALAT1 and H19 are early-stage lncRNA candidates and circRNAs remain preclinical. Despite strong mechanistic evidence, no autophagy-related ncRNA has yet achieved sufficient validation for routine management of patients with myocardial infarction undergoing coronary reperfusion (8, 50, 51, 58, 62–69, 71–76, 78–82, 85–96, 115–125).

### 6.1 Translational challenges and clinical perspectives of ncRNA-based biomarkers and therapeutics in myocardial IR injury

#### 6.1.1 Challenges in translating ncRNA-based therapeutics into clinical practice

Although accumulating experimental evidence suggests that ncRNAs play pivotal roles in regulating autophagy and myocardial IR injury, translating these findings into effective clinical therapies remains challenging. Most current evidence originates from *in vitro* studies and small animal models, whereas robust validation in large multicenter clinical trials is still lacking (115–117).

One of the major barriers is the efficient and targeted delivery of ncRNA therapeutics. Naked miRNAs, lncRNAs, and circRNAs are rapidly degraded by circulating nucleases and exhibit poor cellular uptake, necessitating the use of delivery vehicles such as lipid nanoparticles (LNPs), viral vectors, extracellular vesicles (exosomes), or chemically modified oligonucleotides (115, 118, 119). Although these platforms improve stability and intracellular delivery, they may also induce immunogenicity, systemic toxicity, or unfavorable biodistribution, thereby limiting their clinical applicability (115, 118, 119).

An additional challenge is the limited conservation of many lncRNAs across species. Unlike miRNAs, which are generally highly conserved between rodents and humans, most lncRNAs exhibit relatively poor sequence conservation, distinct genomic organization, and species-specific expression patterns, limiting the direct extrapolation of findings from mouse models to human disease (126–128). Consequently, the biological functions of lncRNAs identified in experimental myocardial IR injury require validation in human cardiac tissues, induced pluripotent stem cell-derived cardiomyocytes, and large-animal models before clinical translation. Furthermore, although LNPs, adeno-associated viral (AAV) vectors, and engineered exosomes have emerged as promising delivery systems for cardiac ncRNA therapeutics, each possesses inherent limitations. LNPs generally exhibit limited cardiac tropism and transient expression, AAV vectors provide long-term transgene expression but are associated with immunogenicity, restricted opportunities for repeated dosing, and potential safety concerns, whereas exosomes offer excellent biocompatibility but remain constrained by challenges in scalable manufacturing, cargo-loading efficiency, and tissue-targeting specificity (126, 129–131).

Another critical challenge is achieving cardiac-specific delivery. Because systemic administration may expose multiple organs to ncRNA therapeutics, unintended modulation of gene expression in non-cardiac tissues can occur. This concern is amplified by the fact that a single ncRNA often regulates numerous target genes and signalling pathways, increasing the potential for off-target effects and adverse biological consequences (115, 118, 119).

In addition, important gaps remain in our understanding of the pharmacokinetics, long-term safety, and optimal dosing strategies of ncRNA-based therapeutics. Future studies should prioritize the development of highly efficient cardiac-targeted delivery systems, minimize off-target toxicity, and validate therapeutic efficacy and safety in large-animal models and well-designed multicentre clinical trials before routine clinical application becomes feasible.

#### 6.1.2 Methodological challenges in developing circulating ncRNAs as reliable biomarkers

The clinical implementation of circulating ncRNAs as diagnostic or prognostic biomarkers for myocardial IR injury also faces substantial methodological and biological challenges. Pre-analytical variables—including sample type (serum versus plasma), blood collection tubes, processing time, storage conditions, freeze–thaw cycles, hemolysis, and RNA extraction procedures—can markedly influence measured ncRNA concentrations and contribute to considerable inter-study variability (83, 132–138).

Analytical variability further complicates biomarker development. Different RNA extraction kits may yield substantially different RNA quantities and quality, while commonly used detection platforms—including quantitative PCR, microarrays, and next-generation sequencing—often produce inconsistent results. Moreover, the absence of universally accepted endogenous reference controls and standardized normalization strategies remains a major obstacle to reproducibility and comparison across laboratories (83, 133, 138).

Biological heterogeneity represents an additional challenge. Circulating ncRNA profiles are influenced by numerous patient-related factors, including age, sex, diabetes mellitus, hypertension, obesity, dyslipidemia, chronic kidney disease, inflammatory disorders, and other cardiovascular comorbidities (83, 133, 137). Furthermore, widely prescribed cardiovascular medications—including statins, angiotensin-converting enzyme inhibitors, angiotensin receptor blockers, *β*-blockers, antiplatelet agents, sodium–glucose cotransporter-2 inhibitors, and glucagon-like peptide-1 receptor agonists—can independently alter circulating ncRNA expression, making it difficult to distinguish disease-specific signatures from treatment-related changes (83, 133, 138).

Additional complexity arises from differences among biological specimens. Serum, plasma, urine, peripheral blood cells, and extracellular vesicles frequently exhibit distinct ncRNA expression profiles, highlighting the importance of selecting appropriate sample sources for specific clinical applications. Consequently, careful patient selection, rigorous control of confounding variables, and standardized experimental workflows are essential when evaluating ncRNAs as biomarkers or therapeutic targets (132–138).

Overall, although circulating ncRNAs remain highly promising biomarkers for myocardial IR injury, their successful clinical translation will require internationally standardized protocols for sample collection, processing, RNA extraction, normalization, and analytical validation. Large prospective multicenter studies are also needed to establish robust reference ranges, determine clinical cut-off values, and validate diagnostic and prognostic performance across diverse patient populations before ncRNA-based biomarkers can be incorporated into routine cardiovascular practice (83, 132–135, 137, 138).

## 7 Conclusion

### 7.1 Mechanistic summary

ncRNAs are central regulators of autophagy during myocardial IR injury and act at multiple stages of the autophagic pathway. At the induction stage, miRNAs regulate upstream nutrient- and energy-sensing pathways and the ULK1/2 complex, thereby determining initiation of autophagy. During autophagosome nucleation, ncRNAs modulate the class III PI3 K complex, particularly ATG14 and Beclin-1, through direct miRNA–mRNA interactions, competitive endogenous RNA networks, transcription-factor binding, and epigenetic regulation. During elongation, miRNAs, lncRNAs, and circRNAs control the ATG12–ATG5–ATG16L1 and LC3 lipidation systems by regulating ATG3, ATG5, ATG7, ATG12, and related proteins. By contrast, the contribution of ncRNAs to autophagosome–lysosome fusion in myocardial IR injury remains poorly defined, although evidence from non-cardiac tissues suggests that ncRNAs may target LAMP1/2, Rab7, STX17, SNAP29, and VAMP7/8.

The functional effects of these regulatory interactions are highly context dependent. Moderate autophagy during ischemia may preserve energy balance, remove damaged cellular components, and support cardiomyocyte survival, whereas excessive or incomplete autophagy during reperfusion may contribute to mitochondrial dysfunction, oxidative stress, apoptosis, and autophagic cell death. Therefore, ncRNAs should not be classified simply as protective or harmful. Their effects depend on the stage of IR, the extent and duration of autophagy, the efficiency of lysosomal degradation, and the specific experimental or clinical setting.

### 7.2 Clinical relevance

The broad involvement of ncRNAs in autophagy gives them considerable potential as biomarkers and therapeutic targets in myocardial IR injury. Circulating miRNAs, lncRNAs, and circRNAs may reflect myocardial stress, cardiomyocyte death, inflammation, mitochondrial damage, and changes in autophagic activity. Their relative stability in blood and extracellular vesicles also supports their possible use in diagnosis, risk stratification, prognosis, and monitoring of treatment response.

Therapeutically, ncRNAs may provide opportunities to fine-tune autophagy rather than completely activate or inhibit it. miRNA mimics, antisense oligonucleotides, lncRNA-targeting approaches, and circRNA-based interventions could be designed to suppress excessive autophagy during reperfusion or enhance adaptive autophagy during ischemia. ncRNA-modified exosomes and other targeted delivery systems may further improve cardiac uptake and reduce systemic exposure. However, because individual ncRNAs frequently regulate several genes and signalling pathways, therapeutic strategies must account for their pleiotropic effects and for the dual role of autophagy in myocardial injury.

### 7.3 Translational limitations

Despite extensive experimental evidence, clinical translation remains limited. Most mechanistic studies have been performed in cultured cardiomyocytes, hypoxia–reoxygenation models, or small animals. These systems do not fully reproduce the complexity of human myocardial IR injury, including differences in ischemic duration, reperfusion timing, age, sex, comorbidities, medication use, and immune responses.

Interpretation is also complicated by inconsistent assessment of autophagy. Increased LC3-II levels or autophagosome abundance may represent either enhanced autophagosome formation or impaired lysosomal degradation. Many studies do not evaluate dynamic autophagic flux using complementary markers such as p62 degradation, lysosomal function, or fusion assays. Consequently, some reported increases in “autophagy” may actually reflect blocked autophagosome clearance.

The delivery of ncRNA-based therapeutics presents additional barriers. Unmodified RNAs are susceptible to nuclease degradation, have limited cellular uptake, and may accumulate in non-cardiac tissues. Lipid nanoparticles, viral vectors, chemically modified oligonucleotides, and extracellular vesicles may improve delivery but can introduce immunogenicity, toxicity, manufacturing, and biodistribution concerns. Off-target effects are particularly relevant because a single ncRNA may regulate multiple transcripts in different tissues.

The use of circulating ncRNAs as biomarkers is similarly constrained by variation in sample type, collection procedures, hemolysis, processing time, storage, RNA extraction, detection platform, and normalization strategy. Patient characteristics and commonly prescribed cardiovascular or metabolic medications may also alter ncRNA expression independently of myocardial ischemia–reperfusion injury. These factors currently limit reproducibility and comparison across studies.

### 7.4 Future perspectives

Future research should focus on defining the temporal and stage-specific functions of ncRNAs during ischemia and reperfusion. Studies should distinguish adaptive autophagy from excessive autophagy and impaired autophagic flux, using standardized experimental models and dynamic measurements of autophagosome formation, lysosomal fusion, and cargo degradation.

Greater attention should be directed toward the late stages of autophagy. In particular, the possible regulation of LAMP2, Rab7, STX17, SNAP29, VAMP7/8, UVRAG, and lysosomal acidification by ncRNAs in myocardial IR injury requires direct experimental validation. Clarifying these mechanisms may reveal whether impaired autophagosome clearance is a major determinant of cardiomyocyte death and identify targets capable of restoring complete autophagic flux.

Integrated multi-omics approaches combining transcriptomics, epigenomics, proteomics, and spatial or single-cell analyses may help identify cell-specific ncRNA networks in cardiomyocytes, fibroblasts, endothelial cells, and immune cells. Large-animal studies and human myocardial samples will be essential for confirming species conservation and clinical relevance. In parallel, cardiac-targeted delivery systems, including engineered extracellular vesicles, ligand-modified nanoparticles, and tissue-selective oligonucleotides, should be optimized for efficacy, safety, and controlled dosing.

For biomarker development, standardized protocols for sample collection, RNA isolation, quality control, normalization, and reporting are urgently needed. Large prospective multicenter studies should determine whether ncRNA signatures provide diagnostic or prognostic value beyond established clinical markers. Ultimately, the greatest therapeutic potential may lie in precisely modulating selected ncRNA–autophagy axes according to disease stage, thereby preserving beneficial autophagy during ischemia while preventing excessive or incomplete autophagy during reperfusion.

## Data Availability

Data sharing is not applicable to this article as no datasets were generated or analyzed during this current study.

## Glossary

AKT: protein kinase B
AMI: acute myocardial infarction
AMPK: adenosine monophosphate-activated protein kinase
APF: Autophagy promoting factor
ACS: acute coronary syndrome
ATG: Autophagy related
ATG5: autophagy Related Gene 5
BECN1: Beclin-1 Gene
CAD: coronary artery disease
CAIF: cardiac autophagy inhibitory factor
ceRNA: competitive endogenous RNA
circRNAs: Circular RNAs
CMA: chaperone-mediated autophagy
DDIT4: DNA damage-inducible transcript 4
Dnmt3B: DNA methyltransferase 3B
H/R injury: hypoxia/reoxygenation injury
HGP: human genome project
HsTnT: high-sensitivity cardiac troponin T
IR injury: Ischemia Reperfusion injury
LAMP: lysosomal membrane protein
LC3: microtubule associated protein 1 light-chain 3
LncRNAs: long non-coding RNAs
MACE: major adverse cardiovascular events
MALAT1: Metastasis Associated Lung Adenocarcinoma Transcript 1
mRNA: messenger RNA
miRNA: microRNA
IR: ischemia-reperfusion
mTOR: mammalian target of rapamycin
ncRNAs: non-coding RNAs
NEAT1: nuclear enriched abundant transcript 1
NGS: next-generation sequencing
NT-proBNP: NT-pro-natriuretic peptide
OGD/R model: oxygen-glucose deprivation and reoxygenation model
PBs (p-bodies): phase-separated bodies
PC1: percutaneous coronary intervention
Pink1: PTEN-induced putative kinase 1
PIP2: phosphatidylinositol (4, 5)-bisphosphate
PIP3: phosphatidylinositol (3–5)-trisphosphate
PI3 K: Phosphatidylinositol 3-kinase
PTEN: Phosphatase and Tensin homolog
RAB7: Ras-related in brain protein
RISC complex: RNA-induced silencing complex
ROS: Reactive Oxygen Species
SGs: stress granules
SNARE: soluble N-ethylmaleimide-sensitive factor attachment protein receptor
SNAP29: Synaptosome-Associated Protein 29
STEMI: ST-elevation myocardial infarction
STX17: Syntaxin 17
ULK1/2: Unc-51-like kinase 1/2
UVRAG: ultraviolet radiation resistance-associated gene protein
VAMP7/8: vesicle-associated membrane protein 7/8
UVRAG: Ultraviolet Radiation Resistance-Associated.
